# Supplementary material for: Area deprivation, screen time and consumption of food and drink high in fat salt and sugar (HFSS) in young people: results from a cross-sectional study in the UK
Source: BMJ Open. 2019 Jun 28;9(6):e027333. doi: 10.1136/bmjopen-2018-027333 (PMC6609085; doi:10.1136/bmjopen-2018-027333)
Supplement: Author's manuscript [file bmjopen-2018-027333.draft_revisions.pdf]

BMJ Open

BMJ Open is committed to open peer review. As part of this commitment we make the peer review history of every article we publish publicly available.

When an article is published we post the peer reviewers' comments and the authors' responses online. We also post the versions of the paper that were used during peer review. These are the versions that the peer review comments apply to.

The versions of the paper that follow are the versions that were submitted during the peer review process. They are not the versions of record or the final published versions. They should not be cited or distributed as the published version of this manuscript.

BMJ Open is an open access journal and the full, final, typeset and author-corrected version of record of the manuscript is available on our site with no access controls, subscription charges or pay-per-view fees (http://bmjopen.bmj.com).

If you have any questions on BMJ Open's open peer review process please email info.bmjopen@bmj.com

BMJ Open

Health Inequalities, Junk Food Marketing and Obesity in Young People: Results of a Cross-Sectional Study

Journal:	*BMJ Open*
Manuscript ID	bmjopen-2018-027333
Article Type:	Research
Date Submitted by the Author:	17-Oct-2018

Complete List of Authors:	Thomas, Fiona; Cancer Research UK, Cancer Prevention<br>Thomas, Christopher; Cancer Research UK, Policy Research Centre for Cancer Prevention<br>Hooper, Lucie; Cancer Research UK, Policy Research Centre for Cancer Prevention<br>Rosenberg, Gillian; Cancer Research UK, Policy Research Centre for Cancer Prevention<br>Vohra, Jyotsna; Cancer Research UK, Cancer Prevention<br>Bauld, Linda; University of Edinburgh, Usher Institute of Population Health Sciences and Informatics

Keywords:	young people, inequalities, consumption, screen time, health knowledge, deprivation

SCHOLARONE™
Manuscripts

Health Inequalities, Junk Food Marketing and Obesity in Young People: Results of a Cross-Sectional Study

Fiona Thomas [1], Christopher Thomas [1], Lucie Hooper [1], Gillian Rosenberg [1], Jyotsna Vohra [1] and Linda Bauld[1, 2]
[1]Cancer Policy Research Centre, Cancer Research UK
[2]Usher Institute, University of Edinburgh

Key words: Obesity, Young people, Inequalities, Consumption, Screen time, Health Knowledge, Deprivation

Correspondence to: Fiona Thomas, Cancer Research UK, Angel Building, 407 St. John Street London, EC1V 4AD

Contact Details:

Fiona Thomas – fiona.thomas@hotmail.com
Christopher Thomas – Christopher.Thomas@cancer.org.uk
Lucie Hooper – Lucie.Hooper@cancer.org.uk
Gillian Rosenberg – Gillian.Rosenberg@cancer.org.uk
Jyotsna Vohra – Jyotsna.Vohra@cancer.org.uk
Linda Bauld – Linda.Bauld@ed.ac.uk

Word Count: 2832

Number of Tables: 4
Number of References: 49

Corresponding Author:

Fiona Thomas

Cancer Policy Research Centre

Cancer Research UK

Angel Building

407 St. John Street

London

EC1V 4AD

United Kingdom

Tel - 020 3469 6357

For peer review only

Email - fiona.thomas@hotmail.com

For peer review only

Abstract

Objectives: To investigate associations between deprivation in young people and consumption of foods high in fat, salt and sugar (HFSS), screen time exposure and health knowledge.

Design: An online cross-sectional survey with 11-19-year olds in the UK, where participants reported consumption behaviours across 13 HFSS and two non-HFSS groups; screen time for commercial television and streaming services; and knowledge of health conditions and their links to obesity.

Setting: United Kingdom

Participants: 3,348 young people aged 11-19 across the United Kingdom (UK).

Main outcome measures: The study assessed the consumption behaviours, commercial screen time exposure and the health knowledge of 3,348 11-19-year olds. Multivariate binary regression analysis, controlling for age and gender, was performed.

Results: Deprivation level was associated with increases in consumption of six of the HFSS products including energy drinks (OR:2.943 / P< 0.001) and sugary drinks (OR:1.938, P< 0.001), and a reduction in consumption in the two non-HFSS products included in the study; fruit (OR:0.668 / P=0.004) and vegetables (OR:0.306 / P< 0.001). Deprivation was associated with high weekly screen time of both television (OR:2.477 / P< 0.001) and streaming (OR:1.679 / P=0.001). Health knowledge was also associated with deprivation. There was lower awareness of the association of obesity and cancer (OR:0.697 / P=0.003) type 2 diabetes (OR:0.64 / P=0.004) and heart disease (OR:0.519 / P< 0.001) in the most deprived.

Conclusions: Young people from the more deprived areas of the UK were more likely to consume of a range of HFSS products, report increased exposure to HFSS advertising and have a poorer awareness of health conditions associated with overweight and obesity. The findings suggest that population level measures addressing childhood obesity should account for consumption patterns among different groups of children and young people and the factors that may influence these.

For peer review only

Strengths and limitations of this study

- The study identified an association between socio-economic deprivation and risk factors that may influence the prevalence of childhood obesity in the UK through a nationally representative sample of young people aged 11-19 across England and each of the devolved nations.
- Data collected for this study was from a single cross-sectional survey, so it is not possible to determine causation between the variables.
- The study could not directly assess exposure to marketing of foods high in fat, salt and sugar (HFSS), although previous research that had explored the relationship between commercial screen time and exposure and found the two to be related, provided us with greater confidence that screen time may be a reliable proxy for marketing exposure under current UK marketing regulations.

For peer review only

'What this paper adds' box

Section 1: What is already known on this topic?

- Obesity is a complex health condition with multiple drivers
- The most deprived groups are more likely to suffer from negative health outcomes, including those caused by higher rates of childhood obesity.
- An association between greater exposure to junk food marketing and obesity has been found in previous research.
- To date, associations between deprivation, HFSS marketing and obesity in young people have not been fully investigated.

Section 2: What this study adds

- Young people living in more deprived areas reported higher levels of weekly screen time exposure from both television and streaming services.
- The most deprived young people were more likely to consume six of thirteen food and drink categories that are high in fat, salt and sugar (HFSS) and were less likely to consume foods in the two non-HFSS categories included in the study.
- There was lower awareness of the association between obesity and relevant health conditions such as heart disease, cancer and type 2 diabetes among the most deprived respondents.
- Future policies and interventions to address childhood obesity should take into account how these might affect young people living in more deprived communities

For peer review only

BACKGROUND

In the UK, around 30% of children are overweight or obese[1] the highest rate of childhood obesity in Europe. This overall figure masks considerable disparities by socioeconomic status. Overweight and obesity prevalence for children in the 10% most deprived areas in England, for example, is more than double that of those who live in the least deprived 10%[2]. Longer term, an obese child is around five times more likely to become an obese adult[3], and there is substantial evidence that obesity in adulthood directly contributes to the development of conditions such as diabetes, coronary heart disease, stroke and 13 different types of cancer[4-8].

Previous studies have provided diverse explanations for the rise in levels of obesity, ranging from genetics, increased calorie intake, an increase in sedentary behaviour or a combination of factors[9-11]. The calorie intake increase is thought to be the most significant influence accounting for this rise, caused by a range of environmental factors[9,12,13], including the role of the marketing and promotion of foods that are high in fat, salt and sugar (HFSS). Marketing of these foods is extensive and delivered through a variety of platforms including television, streaming, price promotions and print media. Studies have identified a substantial expenditure by manufacturers and retailers on the marketing of junk food to children and young adults[14,15], and identified that industry recognises the potential marketing has for influencing consumption choices[16]. The link between marketing and weight outcomes, as well as increased consumption of HFSS products has been highlighted by a number of previous studies[17-23]. Assessing exposure to HFSS marketing via self-reported recall of viewing advertisements does have limitations. Thus some studies have used commercial screen time as a proxy for TV and online marketing exposure[17,21], whereby greater screen time indicates increased exposure to HFSS advertising. Prior content analysis of UK television, where young people make up a large proportion of the audience, highlighted the increased likelihood of HFSS marketing exposure[24], supporting this proxy measure.

Increasingly, there is a need to identify how marketing and promotion affect children in different social groups or those living in more, or less deprived communities. Studies have previously identified an association between socioeconomic status and obesity[25,26]. Highlighting such associations between socioeconomic status and contributing factors to obesity, such as exposure to HFSS advertising, are important in identifying interventions or policy actions that can contribute to addressing the public health problem. Halting or reversing current obesity trends is a current priority for public health policies in the UK[27,28], and globally[29,30].

Hooper et al[31], identified that there is a low level of public awareness of the link between overweight and obesity and resulting preventable health conditions, including cancer. Only 25% of the UK adult population are aware of this link and this lack of awareness is more prevalent in less affluent groups. Other studies have also found an association between greater health awareness and increased support for policy change, particularly for alcohol policy[32,33]. Greater health knowledge may therefore affect how young people view the acceptability of HFSS marketing and also consumption choices.

To date, there is limited research on the association between deprivation, HFSS marketing and obesity. Given these gaps, this study aims to investigate whether such a relationship exists and how it might be influenced by particular mediators such as frequency and duration of exposure to marketing and knowledge and understanding of health risks.

For peer review only

METHODS

Study design

An online cross-sectional survey was conducted between April and June 2017. The survey was developed following cognitive testing with a small sample of young people (n=100) to ensure age and cultural comprehensibility of the questions, some of which were based on well validated questions used in other surveys[21,34-37]. The final survey covered six main themes; exercise levels; food and drink consumption, screen time, recalled marketing exposure, perceptions of marketing and demographic factors.

A total of 3,348 young people, aged 11-19 were recruited by market research company, YouGov, using their in-house panel. YouGov already had data on the children in households of adult in-house panel members. Children over the age of 16 were directly approached and asked if they wished to participate. For those aged under 16, their parents were contacted and asked if their child could participate in the survey. Data collected was weighted by age, gender, ethnicity, region and social grade to be representative of the UK population.

Measures

Deprivation

Level of deprivation was assessed using an area-based measure rather than individual measure of socio-economic status. The Index of Multiple Deprivation (IMD) was coded into five equal quintiles for analysis, ranging from (1) the lowest 20% of deprivation to (5) the highest 20% of respondents. IMD is a measure of the relative deprivation of an area, combining information from seven domains; income deprivation, employment deprivation, education, skills and training deprivation, health deprivation and disability, crime, barriers to housing and services and living environment deprivation[38].

Consumption Behaviours

The survey measured consumption of a range of food and drink products. Participants were asked to report their consumption behaviours from the question 'How often do you usually eat or drink…?' followed by a series of food categories high in fat, salt and sugar including biscuits and cakes, chips, confectionary, crisps, desserts, diet drinks, energy drinks, flavoured yogurts, milk-based drinks, ready meals, sugary drinks, sweetened cereal and take-aways, as well as with healthy items such as fruit and vegetables. These food groups were chosen using previous research on unhealthy products and with reference to the categories included by Public Health England in their sugar reduction programme[39]. Responses were graded on a Likert scale from more than once a day to never, and then converted to binary variables across two coding groups. The groups identified as 'higher' consumption depending on the total calorific content in each food[17,21,39]. The first coding group included sugar sweetened drinks, flavoured yoghurts, confectionary, cakes/biscuits, fruit, vegetables, diet drinks, crisps and desserts where two or more portions a week was considered high consumption. The second coding group included takeaways, ready meals, energy drinks, fried potato products, milk-based drinks and sugar sweetened cereals where one or more portions a week was considered higher consumption. The coding was only calculated for participants who gave an answer, and those who selected 'not sure' were excluded from the final analysis.

Screen Time

Commercial screen time was a variable created in the data set based on responses related to frequency and

For peer review only

duration of exposure to TV and streaming (on demand) services[21,40]. Participants listed the hours spent watching both commercial and non-commercial TV and streaming services. This excluded screen time from computers being used for homework. Non-commercial screen time (which contains no paid for marketing in the UK context) was shown to not be significant in previous analysis of the data[17-19] and was therefore removed. Weekend and week-day viewing was then weighted and turned into a weekly measure and categorised; low (<3 hours per week), medium (3 – 21 hours per week) and high (21 hours or more per week)[21].

Health Knowledge

Health knowledge was assessed using the question 'Which, if any, of the following health conditions do you think can result from being overweight? Please tick all that apply.' Options included answers that were both correct and incorrect to identify the extent of health knowledge. The eight chosen conditions were; cancer, stroke, heart disease, diabetes type 1, diabetes type 2, migraines, chicken pox and flue. From this, the results were coded as a binary variable: 0 – unaware; 1 – aware of the links between certain conditions and being overweight.

Age and gender

Control variables were selected on theoretical importance from a rapid review of the literature[21,37,40-43] and included gender (coded 0 – Male, 1 – Female) and age (11-19 years).

ETHICS

Ethical approval was obtained in January 2018 from the General University Ethics Panel (GUEP) at the University of Stirling.

ANALYSIS

Data were analysed using IBM SPSS Version 23. Multiple multivariate binary regression models were run on the unweighted data to test for associations between deprivation levels and three key behaviours of young people; consumption behaviours, screen time use and health knowledge. The consumption model used the dependent binary variables of food and drink consumption behaviours. Models were run separately for each dependent variable, producing 15 models in total. The screen time model used the dependent variable of categorised reported screen time hours. The health knowledge model used the dependent binary variable of awareness of a health condition and its link to overweight and obesity. This included eight different health conditions, some with identified associations, and some without.

Within each of these models the Index of Multiple Deprivation (IMD) variable was used as an independent variable, with the least deprived quintile as the reference group. Age and gender were included in the models as control variables, as potentially confounding variables.

RESULTS

Sample characteristics

Almost half (49%) of the survey respondents were female and 51% male. The mean age of participants was 14.9 years old (SD = 2.55). The majority (82%) were from white British backgrounds with 18% from

For peer review only

other ethnic groups. The majority of respondents lived in England (82%); 5% of respondents lived in Wales, 8% in Scotland and 3% in Northern Ireland (*Table 1*).

Screen time

Of respondents, there were 31.9% in low, 57.1% in medium and 11.0% in high screen time category for television viewing. For streaming services there was 30.3% in low, 50.7% in medium and 19.0% in high screen time categories.

Deprivation and Consumption Behaviours

The results of the binary logistic regressions showed an association between deprivation and higher consumption behaviours, for a range of HFSS food products (*Table 2*). The most deprived young people were significantly more likely to consume energy drinks (OR= 2.943, P < 0.001), followed by sugary drinks (OR= 1.938, P < 0.001).

In contrast, analysis identified consumption of fruit and vegetables was inversely associated with more deprived groups. Fruit (OR= 0.668, P =0.004) and vegetables (OR= 0.306, P < 0.001) were more likely to be consumed in lower frequency by the most deprived respondents, when compared to the most affluent respondents. Therefore, these young people had a reduced likelihood of consuming the healthier options in higher quantities.

Deprivation and Screen Time

Regression analysis found an association between deprivation in young people and high weekly screen time of both television and streaming (*Table 3*). The model compared 'high' category screen time (21 hours or more a week) to 'medium and low' screen time (less than 21 hours a week) and found those from the most deprived quintile were significantly more likely to be in the high screen time category than the more affluent respondents, for both television (OR= 2.477, P < 0.001) and streaming (OR= 1.679, P = 0.001).

Deprivation and Health Knowledge

The analysis identified an association between deprivation and poorer health knowledge (*Table 4*). Respondents were asked whether eight health conditions (from a pre-existing list) could occur as a result of being overweight or obese. There was significantly poorer awareness of the association between cancer (OR= 0.697, P = 0.003), type 2 diabetes (OR= 0.64, P = 0.004) and heart disease (OR= 0.519, P < 0.001) and obesity for those from the more deprived quintiles. There was also significantly higher association between incorrectly linking type 1diabetes (OR= 1.536, P < 0.001) and obesity in the most deprived quintile, compared to the most affluent quintile.

For peer review only

For peer review only

DISCUSSION

Results from this survey identify a clear association between socio-economic deprivation and risk factors that may influence the prevalence of childhood obesity in the UK. Involving a nationally representative sample of young people aged 11-19 across England and each of the devolved nations, it sought to explore whether young people living in more deprived areas reported knowledge and behaviours that may contribute to obesity. The study found that these young people consumed more foods and beverages high in salt sugar and fat, and were conversely less likely to report consumption of fruit and vegetables. In addition, young people living in more deprived communities spent more time watching commercial broadcast media where they could be exposed to HFSS advertising. Less affluent young people also had lower levels of awareness of the preventable health conditions, including cancer, which can arise as a result of obesity.

These results support findings from previous studies on factors influencing childhood obesity but also provide new evidence on the clustering of these factors amongst less affluent groups. It is well established that there is a clear gradient in overweight and obesity by socio-economic status in both adults and young people, with individuals from less affluent communities more likely to carry excess weight compared to their more affluent neighbours[2,25,26,44-46]. More limited research has explored how eating patterns vary by deprivation in young people. This study adds to existing evidence suggesting that greater HFSS consumption and lower levels of fruit and vegetable consumption are more common in less affluent young people[18,47].

This survey also asked young people about the time they spend watching television and on-demand screening services and calculated ‘screen time’ using an approach employed in previous studies[17,21]. While higher levels of screen time are associated with sedentary behaviour, which may contribute to obesity, they may also suggest greater exposure to broadcast media marketing including of HFSS foods. Previous research has found that children who spent more time watching commercial TV and on demand programmes in the UK are exposed to more HFSS food marketing than those with lower levels of screen time[24,48]. Viewing more HFSS ads on TV and streaming has been associated with higher HFSS consumption, with the difference between a high consumer and a low consumer being at least 520 junk food products a year[17].

We also found that young people living in more deprived areas had lower levels of awareness of the links between overweight and obesity and relevant health conditions. Awareness is relevant because evidence relating to other preventable risk factors (such as smoking and alcohol) suggest that health knowledge is relevant as a preliminary step towards changing behaviour, but also, importantly, understanding and support for policies and interventions that may address key factors that drive consumption including restrictions on marketing and pricing of unhealthy products[32,33,49].

Taken together our findings suggest that inequalities in rates of obesity in young people in the UK may be linked to knowledge and behaviours driven by key aspects of an obesogenic environment. Action to address childhood obesity needs to take into account differential consumption patterns amongst less affluent young people and the factors that may influence these consumption patterns. The introduction of policies and interventions that aim to address these factors, including better information on the health consequences of obesity, reducing exposure to HFSS marketing and other wider population level measures (such as policies to address the price and content of products) should consider and assess their impact on less affluent groups.

Strengths and limitations of the study

For peer review only

This study has a number of limitations. Data are from a single cross-sectional survey, so it is not possible to determine causation. Responses to each of the key topics of interest including food consumption patterns, use of streaming services and TV viewing, and awareness of health conditions linked to obesity were based on self-report and thus subject to mis-reporting or recall bias. The study could not directly assess exposure to HFSS marketing, although our previous research has explored the relationship between commercial screen time and exposure and found the two to be related[17,21], which provides us with greater confidence that screen time may be a reliable proxy for marketing exposure under current UK marketing regulations. Finally, overweight and obesity in young people is driven by a wide range of factors beyond those assessed in this study.

Conclusions and future research

Taken together our findings suggest that inequalities in rates of obesity in young people in the UK may be linked to knowledge and behaviours driven by key aspects of an obesogenic environment. Action to address childhood obesity needs to take into account differential consumption patterns amongst less affluent young people and the factors that may influence these consumption patterns. The introduction of policies and interventions that aim to address these factors, including better information on the health consequences of obesity, reducing exposure to HFSS marketing and other wider population level measures (such as policies to address the price and content of products) should consider and assess their impact on less affluent groups.

Future research should explore in more detail a larger number of factors, including, for example, the affordability and availability of HFSS foods, social norms and the influence of social networks in more deprived communities and how these influence knowledge and behaviour among more deprived young people. In addition, studies should assess the impact of changes to the policy and regulatory environment proposed in the UK and other counties to reduce childhood obesity and how these changes may affect young people living in communities where obesity rates are highest.

FUNDING AND ACKNOWLEDGEMENTS

We are grateful to the Cancer Policy Research Centre, Cancer Research UK for funding the study. The views expressed are those of the researchers and not necessarily those of the funder.

For peer review only

Table 1: Sample Demographics of the UK Representative respondents

Male	11 to 12	11.0%
	13 to 15	16.0%
	16 to 17	12.0%
	18 to 19	12.0%
Female	11 to 12	10.0%
	13 to 15	16.0%
	16 to 17	11.0%
	18 to 19	12.0%
Ethnicity	White	82.0%
	BME	18.0%
IMD	1,2	20.0%
	3,4	20.0%
	5,6	20.0%
	7,8	20.0%
	9,10	20.0%
Region	North East	4.0%
	North West	11.1%
	Yorkshire & Humber	8.5%
	East Midlands	7.3%
	West Midlands	9.3%
	East	9.3%
	London	12.7%
	South East	14.0%
	South West	8.2%
	Wales	4.7%
	Scotland	7.8%
	Northern Ireland	3.1%

For peer review only

For peer review only

Table 2: Consumption behaviours and deprivation. The bolded values indicate significance from the model.

	Consumption Behaviours						
	Descriptive Findings				Logistic Regression / Significance		
	Most Deprived 20%		Least Deprived 20%		Most deprived quintile		
	High Consumption (%)	Low Consumption (%)	High Consumption (%)	Low Consumption (%)	OR:	CI (95%)	P - value
**Biscuits and cakes**	58.4%	41.6%	63.6%	36.4%	0.841	0.638 - 1.038	0.097
**Chips**	72.1%	27.9%	67.5%	32.5%	1.259	0.974 - 1.627	0.079
**Confectionary**	65.3%	34.7%	66.3%	33.7%	0.962	0.751 - 1.232	0.759
**Crisps**	62.8%	37.2%	58.5%	41.5%	1.232	0.965 - 1.572	0.093
**Desserts**	47.1%	52.9%	55.2%	44.8%	**0.732**	**0.576 - 0.930**	**0.011**
**Diet Drinks**	35.7%	64.3%	31.2%	68.8%	1.233	0.959 - 1.585	0.102
**Energy drinks**	15.6%	84.4%	7.0%	93.0%	**2.493**	**1.676 - 3.706**	**0.000**
**Flavoured yogurts**	28.0%	72.0%	27.3%	72.7%	1.061	0.812 - 1.386	0.067
**Fruit**	71.3%	28.7%	78.8%	21.2%	**0.668**	**0.507 - 0.879**	**0.004**
**Milk based drinks**	31.3%	68.7%	22.6%	77.4%	**1.613**	**1.229 - 2.118**	**0.001**
**Ready meals**	64.3%	35.7%	56.3%	43.7%	**1.416**	**1.111 - 1.712**	**0.005**
**Sugary drinks**	41.6%	58.4%	27.2%	72.8%	**1.938**	**1.506 - 2.494**	**0.000**
**Sweetened cereals**	49.6%	50.4%	44.8%	55.2%	1.253	0.986 - 1.593	0.066
**Take-aways**	39.1%	60.9%	25.1%	74.9%	**1.914**	**1.482 - 2.472**	**0.000**
**Vegetables**	78.9%	21.1%	92.4%	7.6%	**0.306**	**0.211 - 0.442**	**0.000**

Table 3: Screen-time behaviour and deprivation. The bolded values indicate significance from the model.

For peer review only

	Screen Time Behaviours								
	Frequency						Logistic Regression / Significance		
	Most Deprived (20%)			Least Deprived (20%)			Most Deprived Quintile		
	Low Viewing %	Medium Viewing %	High Viewing %	Low Viewing %	Medium Viewing %	High Viewing %	OR	CI	P-Value
**Television Screen Time**	25.7%	55.9%	18.4%	31.7%	60.0%	8.3%	**2.477**	**1.697 - 3.614**	**0.000**
**Streaming Screen Time**	28.2%	46.8%	25.0%	33.2%	50.3%	16.5%	**1.679**	**1.234 - 2.283**	**0.001**

For peer review only

Table 4: Health knowledge and deprivation. The bolded values indicate significance from the model.

		Frequency		Logistic Regression / Significance	
	**Overall awareness**	**Most Deprived Awareness %**	**Least Deprived Awareness %**	**OR**	**P Value**
**Cancer Link**	42.0%	36.1%	44.4%	**0.697**	**[0.003]**
**Heart Disease Link**	87.0%	83.5%	90.3%	**0.519**	**[0.000]**
**Stroke Link**	60.0%	62.1%	59.2%	0.862	[0.228]
**Diabetes Type 1 Link**	39.0%	46.1%	36.3%	**1.536**	**[0.000]**
**Diabetes Type 2 Link**	82.0%	78.7%	84.7%	**0.64**	**[0.004]**
**Flu Link**	4.0%	4.1%	2.7%	1.544	[0.196]
**Chicken Pox Link**	1.0%	0.4%	0.7%	0.558	[0.502]
**Migraine Link**	14.0%	11.0%	11.8%	0.907	[0.604]

For peer review only

References

1. National Child Measurement Programme (NCMP): Trends in child BMI. Public Health England; 2017.
2. Patterns and Trends in Child Obesity. 2017. https://www.gov.uk/guidance/phe-data-and-analysis-tools#obesity-diet-and-physical-activity2018).
3. Simmonds M, Llewellyn A, Owen CG, Woolacott N. Predicting adult obesity from childhood obesity: a systematic review and meta-analysis. *Obes Rev* 2016; **17**(2): 95-107.
4. Parkin DM, Boyd L. 8. Cancers attributable to overweight and obesity in the UK in 2010. *Br J Cancer* 2011; **105 Suppl 2**: S34-7.
5. Ades PA, Savage PD. Obesity in coronary heart disease: An unaddressed behavioral risk factor. *Prev Med* 2017; **104**: 117-9.
6. Brown KF, Rumgay H, Dunlop C, et al. The fraction of cancer attributable to modifiable risk factors in England, Wales, Scotland, Northern Ireland, and the United Kingdom in 2015. *Br J Cancer* 2018; **118**(8): 1130-41.
7. Adult obesity and type 2 diabetes. Public Health England; 2014.
8. Organization. WH. Global Health Risks-Mortality and burden of disease attributable to selected major risks

The Lancet 2015.
9. Bleich S, Cutler D, Murray C, Adams A. Why is the developed world obese? *Annu Rev Public Health* 2008; **29**: 273-95.
10. Ebbeling CB, Pawlak DB, Ludwig DS. Childhood obesity: public-health crisis, common sense cure. *Lancet* 2002; **360**(9331): 473-82.
11. Reilly JJ. Obesity in childhood and adolescence: evidence based clinical and public health perspectives. *Postgrad Med J* 2006; **82**(969): 429-37.
12. Afshin A, Penalvo JL, Del Gobbo L, et al. The prospective impact of food pricing on improving dietary consumption: A systematic review and meta-analysis. *PLoS One* 2017; **12**(3): e0172277.
13. Vandevijvere S, Chow CC, Hall KD, Umali E, Swinburn BA. Increased food energy supply as a major driver of the obesity epidemic: a global analysis. *Bull World Health Organ* 2015; **93**(7): 446-56.
14. Alliance OH. Health costs of obesity soaring as junk food companies pour millions into advertising. 2017.
15. Powell L, Harris J, Fox T. Food Marketing Expenditures Aimed at Youth Putting the Numbers in Context. *American Journal of Preventative Medicine* 2014; **45**(4): 453-61.
16. Story M, French S. Food Advertising and Marketing Directed at Children and Adolescents in the US. *Int J Behav Nutr Phys Act* 2004; **1**(1): 3.
17. Thomas C, Hooper L, Petty R, Thomas F, Rosenberg G, Vohra J. 10 Years On: Cancer Research UK, 2018.
18. Thomas C, Hooper L, Petty R, Thomas F, Rosenberg G, Vohra J. Under Pressure: New evidence on young people's broadcast marketing exposure in the UK: Cancer Research UK, 2018.
19. Thomas F, Hooper L, Petty R, Thomas C, Rosenberg G, Vohra J. A Prime Time for Action: New evidence on the link between television and on-demand marketing and obesity: Cancer Research UK, 2018.
20. Boyland EJ, Nolan S, Kelly B, et al. Advertising as a cue to consume: a systematic review and meta-analysis of the effects of acute exposure to unhealthy food and nonalcoholic beverage advertising on intake in children and adults. *Am J Clin Nutr* 2016; **103**(2): 519-33.
21. Scully M, Wakefield M, Niven P, et al. Association between food marketing exposure and adolescents' food choices and eating behaviors. *Appetite* 2012; **58**(1): 1-5.

For peer review only

22. Halford JC, Boyland EJ, Hughes G, Oliveira LP, Dovey TM. Beyond-brand effect of television (TV) food advertisements/commercials on caloric intake and food choice of 5-7-year-old children. *Appetite* 2007; **49**(1): 263-7.
23. National Child Measurement Programme. Changes in children's body mass index between 2006/07 and 2015/16.: Public Health England, 2017.
24. Boyland EJ, Harrold JA, Kirkham TC, Halford JC. The extent of food advertising to children on UK television in 2008. *International Journal of Pediatric Obesity* 2011; **6**(5-6): 455-61.
25. Wang Y. Cross-national comparison of childhood obesity: the epidemic and the relationship between obesity and socioeconomic status. *Int J Epidemiol* 2001; **30**(5): 1129-36.
26. England PH. Severe obesity in ten to eleven year olds reaches record high. In: Mills J, editor.: Public Health England; 2018.
27. A Healthier Future - Scotland's Diet and Healthy Weight Delivery Plan. Edinburgh: Scottish Government, 2018.
28. Department of Health and Social Care: Global Public Health Directorate: Obesity FaN. Childhood Obesity: a plan for action. Chapter 2: HM Government, 2018.
29. Swinburn BA. Obesity prevention: the role of policies, laws and regulations. *Aust New Zealand Health Policy* 2008; **5**: 12.
30. WHO. World Health Organisation: Obesity and Overweight. 2017. http://www.who.int/en/news-room/fact-sheets/detail/obesity-and-overweight (accessed 24.07.18 2018).
31. Hooper L, Anderson A, Birch J, et al. Public awareness and healthcare professional advice for obesity as a risk factor for cancer in the UK: a cross-sectional survey. *Journal of Public Health* 2017.
32. Buykx P, Li J, Lovatt M, et al. An Examination of Public Attitudes Towards Alcohol Policy: University of Sheffield and Cancer Research UK, 2016.
33. Bates S, Holmes J, Gavens L, et al. Awareness of alcohol as a risk factor for cancer is associated with public support for alcohol policies. *BMC Public Health* 2018; **18**(1): 688.
34. England. PH. National Diet and Nutrition Survey. 2016. https://www.gov.uk/government/collections/national-diet-and-nutrition-survey (accessed 03.01.18 2018).
35. Marketing IoS. Food Choice Survey: University of Stirling, 2007.
36. Alcohol (Minimum Unit) Pricing Act 2012. http://www.legislation.gov.uk/asp/2012/4/pdfs/asp_20120004_en.pdf.
37. Morley BC, Scully ML, Niven PH, et al. What factors are associated with excess body weight in Australian secondary school students? *Med J Aust* 2012; **196**(3): 189-92.
38. Government. DfCaL. The English Index of Multiple Deprivation (IMD) - Guidance, 2015.
39. Sugar Reduction: Achieving the 20%: Public Health England, 2017.
40. Dixon HG, Scully ML, Wakefield MA, White VM, Crawford DA. The effects of television advertisements for junk food versus nutritious food on children's food attitudes and preferences. *Soc Sci Med* 2007; **65**(7): 1311-23.
41. Denny S, Lewycka S, Utter J, et al. The association between socioeconomic deprivation and secondary school students' health: findings from a latent class analysis of a national adolescent health survey. *Int J Equity Health* 2016; **15**(1): 109.
42. Nestle M. Food marketing and childhood obesity--a matter of policy. *N Engl J Med* 2006; **354**(24): 2527-9.
43. Cooke LJ, Wardle J. Age and gender differences in children's food preferences. *Br J Nutr* 2005; **93**(5): 741-6.
44. Stamatakis E, Wardle J, Cole TJ. Childhood obesity and overweight prevalence trends in England: evidence for growing socioeconomic disparities. *Int J Obes (Lond)* 2010; **34**(1): 41-7.

For peer review only

45. McLaren L. Socioeconomic status and obesity. *Epidemiol Rev* 2007; **29**: 29-48.
46. Bann D, Johnson W, Li L, Kuh D, Hardy R. Socioeconomic Inequalities in Body Mass Index across Adulthood: Coordinated Analyses of Individual Participant Data from Three British Birth Cohort Studies Initiated in 1946, 1958 and 1970. *PLoS Med* 2017; **14**(1): e1002214.
47. Crawford B, Byun R, Mitchell E, Thompson S, Jalaludin B, Torvaldsen S. Socioeconomic differences in the cost, availability and quality of healthy food in Sydney. *Aust N Z J Public Health* 2017; **41**(6): 567-71.
48. Campbell D. Children seeing up to 12 adverts for junk food an hour on TV, study finds. 2017. https://www.theguardian.com/society/2017/nov/28/children-seeing-up-to-12-adverts-for-junk-food-an-hour-on-tv-study-finds (accessed 23.07.18 2018).
49. Bryant T. Role of knowledge in public health and health promotion policy change. *Health Promot Int* 2002; **17**(1): 89-98.

For peer review only

Area deprivation, screen time and HFSS consumption in young people: Results from a cross sectional study in the UK

Journal:	*BMJ Open*
Manuscript ID	bmjopen-2018-027333.R1
Article Type:	Research
Date Submitted by the Author:	26-Feb-2019
Complete List of Authors:	Thomas, Fiona; Cancer Research UK, Cancer Prevention<br>Thomas, Christopher; Cancer Research UK, Policy Research Centre for Cancer Prevention<br>Hooper, Lucie; Cancer Research UK, Policy Research Centre for Cancer Prevention<br>Rosenberg, Gillian; Cancer Research UK, Policy Research Centre for Cancer Prevention<br>Vohra, Jyotsna; Cancer Research UK, Cancer Prevention<br>Bauld, Linda; University of Edinburgh, Usher Institute of Population Health Sciences and Informatics
**Primary Subject Heading**:	Public health
Secondary Subject Heading:	Nutrition and metabolism, Health policy, Public health
Keywords:	young people, inequalities, consumption, screen time, health knowledge, deprivation

SCHOLARONE™
Manuscripts

Area deprivation, screen time and HFSS consumption in young people: Results from a cross sectional study in the UK

Fiona Thomas [1], Christopher Thomas [1], Lucie Hooper [1], Gillian Rosenberg [1], Jyotsna Vohra [1] and Linda Bauld[1, 2]
[1]Cancer Policy Research Centre, Cancer Research UK
[2]Usher Institute, University of Edinburgh

Key words: Obesity, Young people, Inequalities, Consumption, Screen time, Health Knowledge, Deprivation

Correspondence to: Fiona Thomas, Cancer Research UK, Angel Building, 407 St. John Street London, EC1V 4AD

Contact Details:

Fiona Thomas – fiona.thomas@hotmail.com
Christopher Thomas – Christopher.Thomas@cancer.org.uk
Lucie Hooper – Lucie.Hooper@cancer.org.uk
Gillian Rosenberg – Gillian.Rosenberg@cancer.org.uk
Jyotsna Vohra – Jyotsna.Vohra@cancer.org.uk
Linda Bauld – Linda.Bauld@ed.ac.uk

Word Count: 3096

Number of Tables: 4
Number of References: 51

Corresponding Author:

Fiona Thomas

Cancer Policy Research Centre

Cancer Research UK

Angel Building

407 St. John Street

London

EC1V 4AD

United Kingdom

Tel - 020 3469 6357

Email - fiona.thomas@hotmail.com

For peer review only

Abstract

Objectives: To investigate associations between deprivation in young people and consumption of foods high in fat, salt and sugar (HFSS), screen time exposure and health knowledge.

Design: An online cross-sectional survey with 11-19-year olds in the UK, where participants reported consumption behaviours across 13 HFSS and two non-HFSS groups; screen time for commercial television and streaming services; and knowledge of health conditions and their links to obesity.

Setting: United Kingdom

Participants: 3,348 young people aged 11-19 across the United Kingdom (UK).

Main outcome measures: The study assessed the consumption behaviours, commercial screen time exposure and the health knowledge of 3,348 11-19-year olds. Multivariate binary regression analysis, controlling for age and gender, was performed.

Results: Deprivation level was associated with increases in consumption of six of the HFSS products including energy drinks (OR:2.943 / P< 0.001) and sugary drinks (OR:1.938, P< 0.001), and a reduction in consumption in the two non-HFSS products included in the study; fruit (OR:0.668 / P=0.004) and vegetables (OR:0.306 / P< 0.001). Deprivation was associated with high weekly screen time of both television (OR:2.477 / P< 0.001) and streaming (OR:1.679 / P=0.001). Health knowledge was also associated with deprivation. There was lower awareness of the association of obesity and cancer (OR:0.697 / P=0.003) type 2 diabetes (OR:0.64 / P=0.004) and heart disease (OR:0.519 / P< 0.001) in the most deprived.

Conclusions: Young people from the more deprived areas of the UK were more likely to consume of a range of HFSS products, report increased exposure to HFSS advertising and have a poorer awareness of health conditions associated with overweight and obesity. The findings suggest that population level measures addressing childhood obesity should account for consumption patterns among different groups of children and young people and the factors that may influence these.

For peer review only

Strengths and limitations of this study

- The study identified an association between socio-economic deprivation and risk factors that may influence the prevalence of childhood obesity in the UK through a nationally representative sample of young people aged 11-19 across England and each of the devolved nations.
- Data collected for this study was from a single cross-sectional survey, so it is not possible to determine causation between the variables.
- The study could not directly assess exposure to marketing of foods high in fat, salt and sugar (HFSS), although previous research that had explored the relationship between commercial screen time and exposure and found the two to be related, provided us with greater confidence that screen time may be a reliable proxy for marketing exposure under current UK marketing regulations.

For peer review only

‘What this paper adds’ box

Section 1: What is already known on this topic?

- Obesity is a complex health condition with multiple drivers
- The most deprived groups are more likely to suffer from negative health outcomes, including those caused by higher rates of childhood obesity.
- An association between greater exposure to junk food marketing and obesity has been found in previous research.
- To date, associations between deprivation, HFSS marketing and obesity in young people have not been fully investigated.

Section 2: What this study adds

- Young people living in more deprived areas reported higher levels of weekly screen time exposure from both television and streaming services.
- The most deprived young people were more likely to consume six of thirteen food and drink categories that are high in fat, salt and sugar (HFSS) and were less likely to consume foods in the two non-HFSS categories included in the study.
- There was lower awareness of the association between obesity and relevant health conditions such as heart disease, cancer and type 2 diabetes among the most deprived respondents.
- Future policies and interventions to address childhood obesity should take into account how these might affect young people living in more deprived communities

For peer review only

BACKGROUND

In the UK, around 30% of children are overweight or obese[1] the highest rate of childhood obesity in Europe. This overall figure masks considerable disparities by socioeconomic status. Overweight and obesity prevalence for children in the 10% most deprived areas in England, for example, is more than double that of those who live in the least deprived 10%[2]. Longer term, an obese child is around five times more likely to become an obese adult[3], and there is substantial evidence that obesity in adulthood directly contributes to the development of conditions such as diabetes, coronary heart disease, stroke and 13 different types of cancer[4-8].

Previous studies have provided diverse explanations for the rise in levels of obesity, ranging from genetics, increased calorie intake, an increase in sedentary behaviour or a combination of factors[9-11]. The calorie intake increase is thought to be the most significant influence accounting for this rise, caused by a range of environmental factors[9,12,13], including the role of the marketing and promotion of foods that are high in fat, salt and sugar (HFSS), often referred to as 'junk foods'. Marketing of these foods is extensive and delivered through a variety of platforms including television, streaming, price promotions and print media. Studies have identified a substantial expenditure by manufacturers and retailers on the marketing of junk food to children and young adults[14, 15], and identified that industry recognises the potential marketing has for influencing consumption choices[16]. The link between marketing and weight outcomes, as well as increased consumption of HFSS products has been highlighted by a number of previous studies[17-23]. Assessing exposure to HFSS marketing via self-reported recall of viewing advertisements does have limitations. Thus some studies have used commercial screen time as a proxy for TV and online marketing exposure[17, 21], whereby greater screen time indicates increased exposure to HFSS advertising. Prior content analysis of UK television, where young people make up a large proportion of the audience, highlighted the increased likelihood of HFSS marketing exposure[24], supporting this proxy measure.

Increasingly, there is a need to identify how marketing and promotion affect children in different social groups or those living in more, or less deprived communities. Studies have previously identified an association between socioeconomic status and obesity[25, 26]. Highlighting such associations between socioeconomic status and contributing factors to obesity, such as exposure to HFSS advertising, are important in identifying interventions or policy actions that can contribute to addressing the public health problem. Halting or reversing current obesity trends is a current priority for public health policies in the UK[27, 28], and globally[29, 30].

Hooper et al[31], identified that there is a low level of public awareness of the link between overweight and obesity and resulting preventable health conditions, including cancer. Only 25% of the UK adult population are aware of this link and this lack of awareness is more prevalent in less affluent groups. Other studies have also found an association between greater health awareness and increased support for policy change, particularly for alcohol policy[32, 33]. Greater health knowledge may therefore affect how young people view the acceptability of HFSS marketing and also consumption choices.

To date, there is limited research on the association between deprivation, HFSS marketing and obesity. Given these gaps, this study aims to investigate whether such a relationship exists and how it might be influenced by particular mediators such as frequency and duration of exposure to marketing and knowledge and understanding of health risks.

For peer review only

METHODS

Study design

An online cross-sectional survey was conducted between April and June 2017. The survey was developed following cognitive testing with a small sample of young people (n=100) to ensure age and cultural comprehensibility of the questions, some of which were based on well validated questions used in other surveys[21, 34-37]. The final survey covered six main themes; exercise levels; food and drink consumption, screen time, recalled marketing exposure, perceptions of marketing and demographic factors.

A total of 3,348 young people, aged 11-19 were recruited by market research company, YouGov, using their in-house panel. YouGov already had data on the children in households of adult in-house panel members. Children over the age of 16 were directly approached and asked if they wished to participate. For those aged under 16, their parents were contacted and asked if their child could participate in the survey. Data collected was weighted by age, gender, ethnicity, region and social grade to be representative of the UK population.

Measures

Deprivation

Level of deprivation was assessed using an area-based measure rather than individual measure of socio-economic status. The Index of Multiple Deprivation (IMD) was coded into five equal quintiles for analysis, ranging from (1) the lowest 20% of deprivation to (5) the highest 20% of respondents. IMD is a measure of the relative deprivation of an area, combining information from seven domains; income deprivation, employment deprivation, education, skills and training deprivation, health deprivation and disability, crime, barriers to housing and services and living environment deprivation[38].

Consumption Behaviours

The survey measured consumption of a range of food and drink products. Participants were asked to report their consumption behaviours from the question 'How often do you usually eat or drink…?' followed by a series of food categories high in fat, salt and sugar including biscuits and cakes, chips, confectionary, crisps, desserts, energy drinks, flavoured yogurts, milk-based drinks, ready meals, sugary drinks, sweetened cereal and take-aways, as well as with healthy items such as fruit and vegetables. These food groups were chosen using previous research on unhealthy products and with reference to the categories included by Public Health England in their sugar reduction programme39. Responses were graded on a Likert scale from more than once a day to never, and then converted to binary variables across two coding groups. The groups identified as 'higher' consumption depending on the total calorific content in each food[17, 21, 39]. The first coding group included sugar sweetened drinks, flavoured yoghurts, confectionary, cakes/biscuits, fruit, vegetables, diet drinks, crisps and desserts where two or more portions a week was considered high consumption[39]. The second coding group included takeaways, ready meals, energy drinks, fried potato products, milk-based drinks and sugar sweetened cereals where one or more portions a week was considered higher consumption[39]. The coding was only calculated for participants who gave an answer, and those who selected 'not sure' were excluded from the final analysis.

Screen Time

Commercial screen time was a variable created in the data set based on responses related to frequency and duration of exposure to TV and streaming (on demand) services[21, 40]. Participants listed the hours

spent watching both commercial and non-commercial TV and streaming services. This excluded screen time from computers being used for homework. Non-commercial screen time (which contains no paid for marketing in the UK context) was shown to not be significant in previous analysis of the data[17-19] and was therefore removed. Weekend and week-day viewing was then weighted and turned into a weekly measure and categorised; low (<3 hours per week), medium (3 – 21 hours per week) and high (21 hours or more per week)[21].

Health Knowledge

Health knowledge was assessed using the question 'Which, if any, of the following health conditions do you think can result from being overweight? Please tick all that apply.' Options included answers that were both correct and incorrect to identify the extent of health knowledge. The eight chosen conditions were; cancer, stroke, heart disease, diabetes type 1, diabetes type 2, migraines, chicken pox and flu. From this, the results were coded as a binary variable: 0 – unaware; 1 – aware of the links between certain conditions and being overweight.

Age and gender

Control variables were selected on theoretical importance from a rapid review of the literature[21, 37, 40-43] and included gender (coded 0 – Male, 1 – Female) and age (11-19 years).

Patient and Public Involvement

Prior to the development of the survey qualitative research was carried out by colleagues at the University of Stirling and National Centre for Social Research. This work consulted young people through focus groups, on the design and design and content of the questionnaire. Results of these focus group discussions were published in a Cancer Research UK report *"It's Just There To Trick You"*[44]. This PPI development work involved discussion of relevant research questions related to food and beverage consumption, relevant policy issues (i.e. exposure to food marketing, pricing, availability), use of broadcast media examples and the appropriateness of questions relating to socio-demographic characteristics. Questions included in the resulting survey were then trialled with young people using cognitive interviewing techniques as described above. Survey reports are publicly available on Cancer Policy Research Centre's website.

ETHICS

Ethical approval was obtained in January 2018 from the General University Ethics Panel (GUEP) at the University of Stirling. This ethical approval covered both cognitive testing of the questionnaires and the online surveys. YouGov's staff included lead for ethical and quality assurance, to ensure adherence to best practice throughout testing and data collection. This included ensuring informed consent was obtained from participants, links were provided to relevant support organisations should participants wish to contact them and confidentiality of personal information was assured.

ANALYSIS

Data were analysed using IBM SPSS Version 23. Multiple multivariate binary regression models were run on the unweighted data to test for associations between deprivation levels and three key behaviours of young people; consumption behaviours, screen time use and health knowledge. The consumption model used the dependent binary variables of food and drink consumption behaviours. Models were run separately for each dependent variable, producing 15 models in total. The screen time model used the dependent variable of categorised reported screen time hours. The health knowledge model used the dependent binary variable of awareness of a health condition and its link to

For peer review only

overweight and obesity. This included eight different health conditions, some with identified associations, and some without.

Within each of these models the Index of Multiple Deprivation (IMD) variable was used as an independent variable, with the least deprived quintile as the reference group. Age and gender were included in the models as control variables, as potentially confounding variables.

RESULTS

Sample characteristics

Almost half (49%) of the survey respondents were female and 51% male. The mean age of participants was 14.9 years old (SD = 2.55). The majority (82%) were from white British backgrounds with 18% from other ethnic groups. The majority of respondents lived in England (82%); 5% of respondents lived in Wales, 8% in Scotland and 3% in Northern Ireland (*Table 1*).

Screen time

Of respondents, there were 31.9% in low, 57.1% in medium and 11.0% in high screen time category for television viewing. For streaming services there was 30.3% in low, 50.7% in medium and 19.0% in high screen time categories.

Deprivation and Consumption Behaviours

The results of the binary logistic regressions showed an association between deprivation and higher consumption behaviours, for a range of HFSS food products (*Table 2*). The most deprived young people were significantly more likely to consume energy drinks (OR= 2.943, P < 0.001), followed by sugary drinks (OR= 1.938, P < 0.001).

In contrast, analysis identified consumption of fruit and vegetables was inversely associated with more deprived groups. Fruit (OR= 0.668, P =0.004) and vegetables (OR= 0.306, P < 0.001) were more likely to be consumed in lower frequency by the most deprived respondents, when compared to the most affluent respondents, as per the use of IMD. Therefore, these young people had a reduced likelihood of consuming the healthier options in higher quantities.

Deprivation and Screen Time

Regression analysis found an association between deprivation in young people and high weekly screen time of both television and streaming (*Table 3*). The model compared 'high' category screen time (21 hours or more a week) to 'medium and low' screen time (less than 21 hours a week) and found those from the most deprived quintile were significantly more likely to be in the high screen time category than the more affluent respondents, for both television (OR= 2.477, P < 0.001) and streaming (OR= 1.679, P = 0.001).

Deprivation and Health Knowledge

The analysis identified an association between deprivation and poorer health knowledge (*Table 4*). Respondents were asked whether eight health conditions (from a pre-existing list) could occur as a result of being overweight or obese. There was significantly poorer awareness of the association between cancer (OR= 0.697, P = 0.003), type 2 diabetes (OR= 0.64, P = 0.004) and heart disease (OR= 0.519, P < 0.001) and obesity for those from the more deprived quintiles. There was also significantly higher association between incorrectly linking type 1diabetes (OR= 1.536, P < 0.001) and obesity in the most deprived quintile, compared to the most affluent quintile.

For peer review only

DISCUSSION

Results from this survey identify a clear association between socio-economic deprivation and risk factors that may influence the prevalence of childhood obesity in the UK. Involving a nationally representative sample of young people aged 11-19 across England and each of the devolved nations, it sought to explore whether young people living in more deprived areas reported knowledge and behaviours that may contribute to obesity. The study found that these young people consumed more foods and beverages high in salt sugar and fat, and were conversely less likely to report consumption of fruit and vegetables. In addition, young people living in more deprived communities spent more time watching commercial broadcast media where they could be exposed to HFSS advertising. Young people living in less affluent areas also had lower levels of awareness of the preventable health conditions, including cancer, which can arise as a result of obesity.

These results support findings from previous studies on factors influencing childhood obesity but also provide new evidence on the clustering of these factors amongst less affluent groups. It is well established that there is a clear gradient in overweight and obesity by socio-economic status in both adults and young people, with individuals from less affluent communities more likely to carry excess weight compared to their more affluent neighbours[2, 25, 26, 45-47]. More limited research has explored how eating patterns vary by deprivation in young people. This study adds to existing evidence suggesting that greater HFSS consumption and lower levels of fruit and vegetable consumption are more common in less affluent young people[18, 48].

This survey also asked young people about the time they spend watching television and on-demand screening services and calculated ‘screen time’ using an approach employed in previous studies[17, 21]. While higher levels of screen time are associated with sedentary behaviour, which may contribute to obesity, they may also suggest greater exposure to broadcast media marketing including of HFSS foods. Previous research has found that children who spent more time watching commercial TV and on demand programmes in the UK are exposed to more HFSS food marketing than those with lower levels of screen time[24, 49]. Viewing more HFSS ads on TV and streaming has been associated with higher HFSS consumption, with the difference between a high consumer and a low consumer being at least 520 junk food products a year[17].

We also found that young people living in more deprived areas had lower levels of awareness of the links between overweight and obesity and relevant health conditions. Awareness is relevant because evidence relating to other preventable risk factors (such as smoking and alcohol) suggest that health knowledge is relevant as a preliminary step towards changing behaviour, but also, importantly, understanding and support for policies and interventions that may address key factors that drive consumption including restrictions on marketing and pricing of unhealthy products[32, 33, 50, 51].

Taken together our findings suggest that inequalities in rates of obesity in young people in the UK may be linked to knowledge and behaviours driven by key aspects of an obesogenic environment. Action to address childhood obesity needs to take into account differential consumption patterns amongst less affluent young people and the factors that may influence these consumption patterns. The introduction of policies and interventions that aim to address these factors, including better information on the health consequences of obesity, reducing exposure to HFSS marketing and other wider population level measures (such as policies to address the price and content of products) should consider and assess their impact on less affluent groups.

Strengths and limitations of the study

This study has a number of limitations. Data are from a single cross-sectional survey, so it is not possible to determine causation. The measure of deprivation used was an areas-based measure (IMD) which has limitations, as individual or household deprivation may vary within areas. Young people may have limited knowledge and awareness of different health conditions, for example the distinction

For peer review only

between Type 1 and Type II diabetes and therefore responses to the question relating to health conditions may in part reflect this lack of understanding. Responses to each of the key topics of interest including food consumption patterns, use of streaming services and TV viewing, and awareness of health conditions linked to obesity were based on self-report and thus subject to mis-reporting or recall bias. The study could not directly assess exposure to HFSS marketing, although our previous research has explored the relationship between commercial screen time and exposure and found the two to be related[17, 21], which provides us with greater confidence that screen time may be a reliable proxy for marketing exposure under current UK marketing regulations. Finally, overweight and obesity in young people is driven by a wide range of factors beyond those assessed in this study.

Conclusions and future research

Taken together our findings suggest that inequalities in rates of obesity in young people in the UK may be linked to knowledge and behaviours driven by key aspects of an obesogenic environment. Action to address childhood obesity needs to take into account differential consumption patterns amongst less affluent young people and the factors that may influence these consumption patterns. The introduction of policies and interventions that aim to address these factors, including better information on the health consequences of obesity, reducing exposure to HFSS marketing and other wider population level measures (such as policies to address the price and content of products) should consider and assess their impact on less affluent groups.

Future research should explore in more detail a larger number of factors, including, for example, the affordability and availability of HFSS foods, social norms and the influence of social networks in more deprived communities and how these influence knowledge and behaviour among more deprived young people. In addition, studies should assess the impact of changes to the policy and regulatory environment proposed in the UK and other counties to reduce childhood obesity and how these changes may affect young people living in communities where obesity rates are highest.

FUNDING AND ACKNOWLEDGEMENTS

We are grateful to the Cancer Policy Research Centre, Cancer Research UK for funding the study. The views expressed are those of the researchers and not necessarily those of the funder.

CONTRIBUTORSHIP STATEMENT

Fiona Thomas carried out the majority of the analysis for this publication along with Chris Thomas and Lucie Hooper who also developed the questions for the survey. Gillian Rosenberg contributed to the preparation of the manuscript and analysis plan. Both Jyotsna Vohra and Linda Bauld contributed to the study design and concept and the preparation of the manuscript.

COMPETING INTERESTS

None

DATA SHARING AGREEMENT

Data can be made available on request

For peer review only

Table 1: Sample Demographics of the UK Representative respondents

Male	11 to 12	11.0%
	13 to 15	16.0%
	16 to 17	12.0%
	18 to 19	12.0%
Female	11 to 12	10.0%
	13 to 15	16.0%
	16 to 17	11.0%
	18 to 19	12.0%
Ethnicity	White	82.0%
	BME	18.0%
IMD	1,2	20.0%
	3,4	20.0%
	5,6	20.0%
	7,8	20.0%
	9,10	20.0%
Region	North East	4.0%
	North West	11.1%
	Yorkshire & Humber	8.5%
	East Midlands	7.3%
	West Midlands	9.3%
	East	9.3%
	London	12.7%
	South East	14.0%
	South West	8.2%
	Wales	4.7%
	Scotland	7.8%
	Northern Ireland	3.1%

For peer review only

Table 2: Consumption behaviours and deprivation. The bolded values indicate significance from the model.

	Consumption Behaviours						
	Descriptive Findings				Logistic Regression / Significance		
	Most Deprived 20%		Least Deprived 20%		Most deprived quintile		
	High Consumption (%)	Low Consumption (%)	High Consumption (%)	Low Consumption (%)	OR:	CI (95%)	P - value
**Biscuits and cakes**	58.4%	41.6%	63.6%	36.4%	0.841	0.638 - 1.038	0.097
**Chips**	72.1%	27.9%	67.5%	32.5%	1.259	0.974 - 1.627	0.079
**Confectionary**	65.3%	34.7%	66.3%	33.7%	0.962	0.751 - 1.232	0.759
**Crisps**	62.8%	37.2%	58.5%	41.5%	1.232	0.965 - 1.572	0.093
**Desserts**	47.1%	52.9%	55.2%	44.8%	**0.732**	**0.576 - 0.930**	**0.011**
**Diet Drinks**	35.7%	64.3%	31.2%	68.8%	1.233	0.959 - 1.585	0.102
**Energy drinks**	15.6%	84.4%	7.0%	93.0%	**2.493**	**1.676 - 3.706**	**0.000**
**Flavoured yogurts**	28.0%	72.0%	27.3%	72.7%	1.061	0.812 - 1.386	0.067
**Fruit**	71.3%	28.7%	78.8%	21.2%	**0.668**	**0.507 - 0.879**	**0.004**
**Milk based drinks**	31.3%	68.7%	22.6%	77.4%	**1.613**	**1.229 - 2.118**	**0.001**
**Ready meals**	64.3%	35.7%	56.3%	43.7%	**1.416**	**1.111 - 1.712**	**0.005**
**Sugary drinks**	41.6%	58.4%	27.2%	72.8%	**1.938**	**1.506 - 2.494**	**0.000**
**Sweetened cereals**	49.6%	50.4%	44.8%	55.2%	1.253	0.986 - 1.593	0.066
**Take-aways**	39.1%	60.9%	25.1%	74.9%	**1.914**	**1.482 - 2.472**	**0.000**
**Vegetables**	78.9%	21.1%	92.4%	7.6%	**0.306**	**0.211 - 0.442**	**0.000**

For peer review only

Table 3: Screen-time behaviour and deprivation. The bolded values indicate significance from the model.

	Screen Time Behaviours								
	Frequency						Logistic Regression / Significance		
	Most Deprived (20%)			Least Deprived (20%)			Most Deprived Quintile		
	Low Viewing %	Medium Viewing %	High Viewing %	Low Viewing %	Medium Viewing %	High Viewing %	OR	CI	P-Value
**Television Screen Time**	25.7%	55.9%	18.4%	31.7%	60.0%	8.3%	**2.477**	**1.697 - 3.614**	**0.000**
**Streaming Screen Time**	28.2%	46.8%	25.0%	33.2%	50.3%	16.5%	**1.679**	**1.234 - 2.283**	**0.001**

For peer review only

Table 4: Health knowledge and deprivation. The bolded values indicate significance from the model.

	Overall awareness	Frequency		Logistic Regression / Significance	
		Most Deprived Awareness %	Least Deprived Awareness %	OR	P Value
**Cancer Link**	42.0%	36.1%	44.4%	**0.697**	**[0.003]**
**Heart Disease Link**	87.0%	83.5%	90.3%	**0.519**	**[0.000]**
**Stroke Link**	60.0%	62.1%	59.2%	0.862	[0.228]
**Diabetes Type 1 Link**	39.0%	46.1%	36.3%	**1.536**	**[0.000]**
**Diabetes Type 2 Link**	82.0%	78.7%	84.7%	**0.64**	**[0.004]**
**Flu Link**	4.0%	4.1%	2.7%	1.544	[0.196]
**Chicken Pox Link**	1.0%	0.4%	0.7%	0.558	[0.502]
**Migraine Link**	14.0%	11.0%	11.8%	0.907	[0.604]

For peer review only

References:

1. National Child Measurement Programme (NCMP): Trends in child BMI. Public Health England; 2017.
2. Patterns and Trends in Child Obesity. 2018. https://www.gov.uk/guidance/phe-data-and-analysis-tools#obesity-diet-and-physical-activity2018).
3. Simmonds M, Llewellyn A, Owen CG, Woolacott N. Predicting adult obesity from childhood obesity: a systematic review and meta-analysis. *Obes Rev* 2016; **17**(2): 95-107.
4. Parkin DM, Boyd, L. 8. Cancers attributable to overweight and obesity in the UK in 2010. *Br Journal of Cancer* 2011; **105 Suppl 2**: S34-7.
5. Ades PA, Savage PD. Obesity in coronary heart disease: An unaddressed behavioural risk factor. *Prev Med* 2017; **104**: 117-9
6. Brown KF, Rumgay H, Dunlop C, et al. The fraction of cancer attributable to modifiable risk factors in England, Wales, Scotland, Northern Ireland, and the United Kingdom in 2015. *Br J Cancer* 2018; **118**(8): 1130-41.
7. Adult obesity and type 2 diabetes. Public Health England; 2014.
8. World Health Organisation. Global Health Risks-Mortality and burden of disease attributable to selected major risks. *The Lancet* 2015.
9. Bleich S, Cutler D, Murray C, Adams A. Why is the developed world obese? *Annu Rev Public Health* 2008; **29**: 273-95
10. Ebbeling CB, Pawlak DB, Ludwig DS. Childhood obesity: public-health crisis, common sense cure. *Lancet* 2002; **360**(9331): 473-82
11. Reilly JJ. Obesity in childhood and adolescence: evidence based clinical and public health perspectives. *Postgrad Med J* 2006; **82**(969): 429-37
12. Afshin A, Penalvo JL, Del Gobbo L, et al. The prospective impact of food pricing on improving dietary consumption: A systematic review and meta-analysis. *PLoS One* 2017; **12**(3): e0172277.
13. Vandevijvere S, Chow CC, Hall KD, Umali E, Swinburn BA. Increased food energy supply as a major driver of the obesity epidemic: a global analysis. *Bull World Health Organ* 2015; **93**(7): 446-56
14. Obesity Health Alliance. Health costs of obesity soaring as junk food companies pour millions into advertising. 2017
15. Powell L, Harris J, Fox T. Food Marketing Expenditures Aimed at Youth Putting the Numbers in Context. *American Journal of Preventative Medicine* 2014; **45**(4): 453-61.
16. Story M, French S. Food Advertising and Marketing Directed at Children and Adolescents in the US. *Int J Behav Nutr Phys Act* 2004; **1**(1): 3.
17. Thomas C, Hooper L, Petty R, Thomas F, Rosenberg G, Vohra J. 10 Years On: Cancer Research UK, 2018.
18. Thomas C, Hooper L, Petty R, Thomas F, Rosenberg G, Vohra J. Under Pressure: New evidence on young people's broadcast marketing exposure in the UK: Cancer Research UK, 2018.
19. Thomas F, Hooper L, Petty R, Thomas C, Rosenberg G, Vohra J. A Prime Time for Action: New evidence on the link between television and on-demand marketing and obesity: Cancer Research UK, 2018.
20. Boyland EJ, Nolan S, Kelly B, et al. Advertising as a cue to consume: a systematic review and meta-analysis of the effects of acute exposure to unhealthy food and nonalcoholic beverage advertising on intake in children and adults. *Am J Clin Nutr* 2016; **103**(2): 519-33.

For peer review only

21. Scully M, Wakefield M, Niven P, et al. Association between food marketing exposure and adolescents' food choices and eating behaviors. *Appetite* 2012; **58**(1): 1-5
22. Halford JC, Boyland EJ, Hughes G, Oliveira LP, Dovey TM. Beyond-brand effect of television (TV) food advertisements/commercials on caloric intake and food choice of 5-7-year-old children. *Appetite* 2007; **49**(1): 263-7
23. National Child Measurement Programme. Changes in children's body mass index between 2006/07 and 2015/16.: Public Health England, 2017
24. Boyland EJ, Harrold JA, Kirkham TC, Halford JC. The extent of food advertising to children on UK television in 2008. *International Journal of Pediatric Obesity* 2011; **6**(5-6): 455-61.
25. Wang Y. Cross-national comparison of childhood obesity: the epidemic and the relationship between obesity and socioeconomic status. *Int J Epidemiol* 2001; **30**(5): 1129-36.
26. England PH. Severe obesity in ten to eleven year olds reaches record high. In: Mills J, editor.: Public Health England; 2018
27. A Healthier Future - Scotland's Diet and Healthy Weight Delivery Plan. Edinburgh: Scottish Government, 2018.
28. Department of Health and Social Care: Global Public Health Directorate: Obesity FaN. Childhood Obesity: a plan for action. Chapter 2: HM Government, 2018
29. Swinburn BA. Obesity prevention: the role of policies, laws and regulations. *Aust New Zealand Health Policy* 2008; **5**: 12
30. WHO. World Health Organisation: Obesity and Overweight. 2017. http://www.who.int/en/news-room/fact-sheets/detail/obesity-and-overweight (accessed 24.07.18 2018).
31. Hooper L, Anderson A, Birch J, et al. Public awareness and healthcare professional advice for obesity as a risk factor for cancer in the UK: a cross-sectional survey. *Journal of Public Health* 2017.
32. Buykx P, Li J, Lovatt M, et al. An Examination of Public Attitudes Towards Alcohol Policy: University of Sheffield and Cancer Research UK, 2016
33. Bates S, Holmes J, Gavens L, et al. Awareness of alcohol as a risk factor for cancer is associated with public support for alcohol policies. *BMC Public Health* 2018; **18**(1): 688
34. Public Health England. National Diet and Nutrition Survey. 2016. https://www.gov.uk/government/collections/national-diet-and-nutrition-survey (accessed 03.01.18 2018)
35. Marketing IoS. Food Choice Survey: University of Stirling, 2007
36. Alcohol (Minimum Unit) Pricing Act 2012. http://www.legislation.gov.uk/asp/2012/4/pdfs/asp_20120004_en.pdf
37. Morley BC, Scully ML, Niven PH, et al. What factors are associated with excess body weight in Australian secondary school students? *Med J Aust* 2012; **196**(3): 189-92.
38. Department for Communities and Local Government. The English Index of Multiple Deprivation (IMD) - Guidance, 2015
39. Sugar Reduction: Achieving the 20%: Public Health England, 2017
40. Dixon HG, Scully ML, Wakefield MA, White VM, Crawford DA. The effects of television advertisements for junk food versus nutritious food on children's food attitudes and preferences. *Soc Sci Med* 2007; **65**(7): 1311-23.
41. Denny S, Lewycka S, Utter J, et al. The association between socioeconomic deprivation and secondary school students' health: findings from a latent class analysis of a national adolescent health survey. *Int J Equity Health* 2016; **15**(1): 109
42. Nestle M. Food marketing and childhood obesity--a matter of policy. *N Engl J Med* 2006; **354**(24): 2527-9.

For peer review only

43. Cooke LJ, Wardle J. Age and gender differences in children's food preferences. *Br J Nutr* 2005; **93**(5): 741-6.
44. MacGregor, A., Bicquelet, A., Lepps. H., Porter, L., Eadie, D., McKell, J., MacKintosh, A A., Thomas, C., Hooper, L and Vohra, J (2016) *""It's just there to trick you": A qualitative study of 11-19 year olds perceptions of food and drink marketing."* ScotCen Social Research; NatCen Social Research; Institute for Social Marketing, University of Stirling; and Policy Research Centre for Cancer Prevention, Cancer Research UK.
45. Stamatakis E, Wardle J, Cole TJ. Childhood obesity and overweight prevalence trends in England: evidence for growing socioeconomic disparities. *Int J Obes (Lond)* 2010; **34**(1): 41-7
46. McLaren L. Socioeconomic status and obesity. *Epidemiol Rev* 2007; **29**: 29-48
47. Bann D, Johnson W, Li L, Kuh D, Hardy R. Socioeconomic Inequalities in Body Mass Index across Adulthood: Coordinated Analyses of Individual Participant Data from Three British Birth Cohort Studies Initiated in 1946, 1958 and 1970. *PLoS Med* 2017; **14**(1): e1002214
48. Crawford B, Byun R, Mitchell E, Thompson S, Jalaludin B, Torvaldsen S. Socioeconomic differences in the cost, availability and quality of healthy food in Sydney. *Aust N Z J Public Health* 2017; **41**(6): 567-71
49. Campbell D. Children seeing up to 12 adverts for junk food an hour on TV, study finds. 2017. https://www.theguardian.com/society/2017/nov/28/children-seeing-up-to-12-adverts-for-junk-food-an-hour-on-tv-study-finds (accessed 23.07.18 2018)
50. Bryant T. Role of knowledge in public health and health promotion policy change. *Health Promot Int* 2002; **17**(1): 89-98
51. Raphael D and Bryant T (2006) The state's role in promoting population health: Public health concerns in Canada, USA, UK and Sweden. *Health Policy.* 78 (1) 39 - 55

For peer review only

STROBE Statement—Checklist of items that should be included in reports of ***cross-sectional studies***

	Item No	Recommendation	Page No.
**Title and abstract**	1	(*a*) Indicate the study's design with a commonly used term in the title or the abstract	1
		(*b*) Provide in the abstract an informative and balanced summary of what was done and what was found	2
**Introduction**			
Background/rationale	2	Explain the scientific background and rationale for the investigation being reported	5
Objectives	3	State specific objectives, including any prespecified hypotheses	5
**Methods**			
Study design	4	Present key elements of study design early in the paper	6
Setting	5	Describe the setting, locations, and relevant dates, including periods of recruitment, exposure, follow-up, and data collection	6
Participants	6	(*a*) Give the eligibility criteria, and the sources and methods of selection of participants	6
Variables	7	Clearly define all outcomes, exposures, predictors, potential confounders, and effect modifiers. Give diagnostic criteria, if applicable	6
Data sources/ measurement	8*	For each variable of interest, give sources of data and details of methods of assessment (measurement). Describe comparability of assessment methods if there is more than one group	6
Bias	9	Describe any efforts to address potential sources of bias	6
Study size	10	Explain how the study size was arrived at	6
Quantitative variables	11	Explain how quantitative variables were handled in the analyses. If applicable, describe which groupings were chosen and why	6, 7
Statistical methods	12	(*a*) Describe all statistical methods, including those used to control for confounding	6, 7
		(*b*) Describe any methods used to examine subgroups and interactions	7
		(*c*) Explain how missing data were addressed	Not recorded for this study as online survey was used
		(*d*) If applicable, describe analytical methods taking account of sampling strategy	6
		(*e*) Describe any sensitivity analyses	NR
**Results**			
Participants	13*	(a) Report numbers of individuals at each stage of study—eg numbers potentially eligible, examined for eligibility, confirmed eligible, included in the study, completing follow-up, and analysed	8

For peer review only - http://bmjopen.bmj.com/site/about/guidelines.xhtml

		(b) Give reasons for non-participation at each stage	This information was not collected.
		(c) Consider use of a flow diagram	-
Descriptive data	14*	(a) Give characteristics of study participants (eg demographic, clinical, social) and information on exposures and potential confounders	8, 11
		(b) Indicate number of participants with missing data for each variable of interest	6
Outcome data	15*	Report numbers of outcome events or summary measures	8
Main results	16	(*a*) Give unadjusted estimates and, if applicable, confounder-adjusted estimates and their precision (eg, 95% confidence interval). Make clear which confounders were adjusted for and why they were included	12,13,14
		(*b*) Report category boundaries when continuous variables were categorized	12,13,14
		(*c*) If relevant, consider translating estimates of relative risk into absolute risk for a meaningful time period	NR
Other analyses	17	Report other analyses done—eg analyses of subgroups and interactions, and sensitivity analyses	NR
**Discussion**			
Key results	18	Summarise key results with reference to study objectives	8
Limitations	19	Discuss limitations of the study, taking into account sources of potential bias or imprecision. Discuss both direction and magnitude of any potential bias	9,10
Interpretation	20	Give a cautious overall interpretation of results considering objectives, limitations, multiplicity of analyses, results from similar studies, and other relevant evidence	9,10
Generalisability	21	Discuss the generalisability (external validity) of the study results	9,10
**Other information**			
Funding	22	Give the source of funding and the role of the funders for the present study and, if applicable, for the original study on which the present article is based	10

*Give information separately for exposed and unexposed groups.

Note: An Explanation and Elaboration article discusses each checklist item and gives methodological background and published examples of transparent reporting. The STROBE checklist is best used in conjunction with this article (freely available on the Web sites of PLoS Medicine at http://www.plosmedicine.org/, Annals of Internal Medicine at http://www.annals.org/, and Epidemiology at http://www.epidem.com/). Information on the STROBE Initiative is available at www.strobe-statement.org.

For peer review only

BMJ Open

Area deprivation, screen time and consumption of food and drink high in fat salt and sugar (HFSS) in young people: Results from a cross sectional study in the UK

Journal:	*BMJ Open*
Manuscript ID	bmjopen-2018-027333.R2
Article Type:	Research
Date Submitted by the Author:	02-May-2019
Complete List of Authors:	Thomas, Fiona; Cancer Research UK, Cancer Prevention<br>Thomas, Christopher; Cancer Research UK, Policy Research Centre for Cancer Prevention<br>Hooper, Lucie; Cancer Research UK, Policy Research Centre for Cancer Prevention<br>Rosenberg, Gillian; Cancer Research UK, Policy Research Centre for Cancer Prevention<br>Vohra, Jyotsna; Cancer Research UK, Cancer Prevention<br>Bauld, Linda; University of Edinburgh, Usher Institute of Population Health Sciences and Informatics
**Primary Subject Heading**:	Public health
Secondary Subject Heading:	Nutrition and metabolism, Health policy, Public health
Keywords:	young people, inequalities, consumption, screen time, health knowledge, deprivation

SCHOLARONE™
Manuscripts

Area deprivation, screen time and consumption of food and drink high in fat salt and sugar (HFSS) in young people: Results from a cross sectional study in the UK

Fiona Thomas [1], Christopher Thomas [1], Lucie Hooper [1], Gillian Rosenberg [1], Jyotsna Vohra [1] and Linda Bauld[1, 2]
[1]Cancer Policy Research Centre, Cancer Research UK
[2]Usher Institute, University of Edinburgh

Key words: Obesity, Young people, Inequalities, Consumption, Screen time, Health Knowledge, Deprivation

Correspondence to: Fiona Thomas, Cancer Research UK, Angel Building, 407 St. John Street London, EC1V 4AD

Contact Details:

Fiona Thomas – fiona.thomas@hotmail.com
Christopher Thomas – Christopher.Thomas@cancer.org.uk
Lucie Hooper – Lucie.Hooper@cancer.org.uk
Gillian Rosenberg – Gillian.Rosenberg@cancer.org.uk
Jyotsna Vohra – Jyotsna.Vohra@cancer.org.uk
Linda Bauld – Linda.Bauld@ed.ac.uk

Word Count: 3191

Number of Tables: 4
Number of References: 54

Corresponding Author:

Fiona Thomas

Cancer Policy Research Centre

Cancer Research UK

Angel Building

407 St. John Street

London

EC1V 4AD

United Kingdom

Tel - 020 3469 6357

Email - fiona.thomas@hotmail.com

For peer review only

Abstract

Objectives: To investigate associations between deprivation in young people and consumption of foods high in fat, salt and sugar (HFSS), screen time exposure and health knowledge.

Design: An online cross-sectional survey with 11-19-year olds in the UK, where participants reported consumption behaviours across 13 HFSS and two non-HFSS groups; screen time for commercial television and streaming services; and knowledge of health conditions and their links to obesity.

Setting: United Kingdom

Participants: 3,348 young people aged 11-19 across the United Kingdom (UK).

Main outcome measures: The study assessed the consumption behaviours, commercial screen time exposure and the health knowledge of 3,348 11-19-year olds. Multivariate binary regression analysis, controlling for age and gender, was performed.

Results: Deprivation level was associated with increases in consumption of six of the HFSS products including energy drinks (OR:2.943 / P< 0.001) and sugary drinks (OR:1.938, P< 0.001), and a reduction in consumption in the two non-HFSS products included in the study; fruit (OR:0.668 / P=0.004) and vegetables (OR:0.306 / P< 0.001). Deprivation was associated with high weekly screen time of both television (OR:2.477 / P< 0.001) and streaming (OR:1.679 / P=0.001). Health knowledge was also associated with deprivation. There was lower awareness of the association of obesity and cancer (OR:0.697 / P=0.003) type 2 diabetes (OR:0.64 / P=0.004) and heart disease (OR:0.519 / P< 0.001) in the most deprived.

Conclusions: Young people from the more deprived areas of the UK were more likely to consume of a range of HFSS products, report increased exposure to HFSS advertising and have a poorer awareness of health conditions associated with overweight and obesity. The findings suggest that population level measures addressing childhood obesity should account for consumption patterns among different groups of children and young people and the factors that may influence these.

For peer review only

Strengths and limitations of this study

- The study identified an association between socio-economic deprivation and risk factors that may influence the prevalence of childhood obesity in the UK through a nationally representative sample of young people aged 11-19 across England and each of the devolved nations.
- Data collected for this study was from a single cross-sectional survey, so it is not possible to determine causation between the variables.
- The study could not directly assess exposure to marketing of foods high in fat, salt and sugar (HFSS), although previous research that had explored the relationship between commercial screen time and exposure and found the two to be related, provided us with greater confidence that screen time may be a reliable proxy for marketing exposure under current UK marketing regulations.

For peer review only

BACKGROUND

In the UK, around 30% of children are overweight or obese[1] the highest rate of childhood obesity in Europe. This overall figure masks considerable disparities by socioeconomic status. Overweight and obesity prevalence for children in the 10% most deprived areas in England, for example, is more than double that of those who live in the least deprived 10%[2]. Longer term, an obese child is around five times more likely to become an obese adult[3], and there is substantial evidence that obesity in adulthood directly contributes to the development of conditions such as diabetes, coronary heart disease, stroke and 13 different types of cancer[4-8].

Previous studies have provided diverse explanations for the rise in levels of obesity, ranging from genetics, increased calorie intake, an increase in sedentary behaviour or a combination of factors[9-11]. The calorie intake increase is thought to be the most significant influence accounting for this rise, caused by a range of environmental factors[9,12,13], including the role of the marketing and promotion of foods that are high in fat, salt and sugar (HFSS), often referred to as 'junk foods'. Marketing of these foods is extensive and delivered through a variety of platforms including television, streaming, price promotions and print media. Studies have identified a substantial expenditure by manufacturers and retailers on the marketing of junk food to children and young adults[14, 15], and identified that industry recognises the potential marketing has for influencing consumption choices[16]. The link between marketing and weight outcomes, as well as increased consumption of HFSS products has been highlighted by a number of previous studies[17-23]. Assessing exposure to HFSS marketing via self-reported recall of viewing advertisements does have limitations. Thus some studies have used commercial screen time as a proxy for TV and online marketing exposure[17, 21], whereby greater screen time indicates increased exposure to HFSS advertising. Prior content analysis of UK television, where young people make up a large proportion of the audience, highlighted the increased likelihood of HFSS marketing exposure[24], supporting this proxy measure.

Increasingly, there is a need to identify how marketing and promotion affect children in different social groups or those living in more, or less deprived communities. Studies have previously identified an association between socioeconomic status and obesity[25, 26]. This association is likely to be caused by a wide variety of factors including the pricing and availability of particular products in a locality[27], understanding and awareness of dietary factors and weight and social norms[28, 29]. However, further research is required to understand the inter-relationship of these factors and also other drivers of consumption including exposure to HFSS advertising. This type of research is important to identify interventions or policy actions that can contribute to addressing overweight and obesity. Halting or reversing current obesity trends is a current priority for public health policies in the UK[30, 31], and globally[32,33].

Hooper et al[34], identified that there is a low level of public awareness of the link between overweight and obesity and resulting preventable health conditions, including cancer. Only 25% of the UK adult population are aware of this link and this lack of awareness is more prevalent in less affluent groups. Other studies have also found an association between greater health awareness and increased support for policy change, particularly for alcohol policy[35, 36]. Greater health knowledge may therefore affect how young people view the acceptability of HFSS marketing and also consumption choices.

To date, there is limited research on the association between deprivation, HFSS marketing and obesity. Given these gaps, this study aims to investigate whether such a relationship exists and how it might be influenced by particular mediators such as frequency and duration of exposure to marketing and knowledge and understanding of health risks.

For peer review only

METHODS

Study design

An online cross-sectional survey was conducted between April and June 2017. The survey was developed following cognitive testing with a small sample of young people (n=100) to ensure age and cultural comprehensibility of the questions, some of which were based on well validated questions used in other surveys[21, 37-40]. The final survey covered six main themes; exercise levels; food and drink consumption, screen time, recalled marketing exposure, perceptions of marketing and demographic factors.

A total of 3,348 young people, aged 11-19 were recruited by market research company, YouGov, using their in-house panel. YouGov already had data on the children in households of adult in-house panel members. Children over the age of 16 were directly approached and asked if they wished to participate. For those aged under 16, their parents were contacted and asked if their child could participate in the survey. Data collected was weighted by age, gender, ethnicity, region and social grade to be representative of the UK population.

Measures

Deprivation

Level of deprivation was assessed using an area-based measure rather than individual measure of socio-economic status. The Index of Multiple Deprivation (IMD) was coded into five equal quintiles for analysis, ranging from (1) the lowest 20% of deprivation to (5) the highest 20% of respondents. IMD is a measure of the relative deprivation of an area, combining information from seven domains; income deprivation, employment deprivation, education, skills and training deprivation, health deprivation and disability, crime, barriers to housing and services and living environment deprivation[41].

Consumption Behaviours

The survey measured consumption of a range of food and drink products. Participants were asked to report their consumption behaviours from the question 'How often do you usually eat or drink…?' followed by a series of food categories high in fat, salt and sugar including biscuits and cakes, chips, confectionary, crisps, desserts, energy drinks, flavoured yogurts, milk-based drinks, ready meals, sugary drinks, sweetened cereal and take-aways, as well as with healthy items such as fruit and vegetables. These food groups were chosen using previous research on unhealthy products and with reference to the categories included by Public Health England in their sugar reduction programme[42]. Responses were graded on a Likert scale from more than once a day to never, and then converted to binary variables across two coding groups. The groups identified as 'higher' consumption depending on the total calorific content in each food[17, 21, 42]. The first coding group included sugar sweetened drinks, flavoured yoghurts, confectionary, cakes/biscuits, fruit, vegetables, diet drinks, crisps and desserts where two or more portions a week was considered high consumption[42]. The second coding group included takeaways, ready meals, energy drinks, fried potato products, milk-based drinks and sugar sweetened cereals where one or more portions a week was considered higher consumption[42]. The coding was only calculated for participants who gave an answer, and those who selected 'not sure' were excluded from the final analysis.

Screen Time

Commercial screen time was a variable created in the data set based on responses related to frequency and duration of exposure to TV and streaming (on demand) services[21, 43]. Participants listed the hours

spent watching both commercial and non-commercial TV and streaming services. This excluded screen time from computers being used for homework. Non-commercial screen time (which contains no paid for marketing in the UK context) was shown to not be significant in previous analysis of the data[17-19] and was therefore removed. Weekend and week-day viewing was then weighted and turned into a weekly measure and categorised; low (<3 hours per week), medium (3 – 21 hours per week) and high (21 hours or more per week)[21].

Health Knowledge

Health knowledge was assessed using the question 'Which, if any, of the following health conditions do you think can result from being overweight? Please tick all that apply.' Options included answers that were both correct and incorrect to identify the extent of health knowledge. The eight chosen conditions were; cancer, stroke, heart disease, diabetes type 1, diabetes type 2, migraines, chicken pox and flu. From this, the results were coded as a binary variable: 0 – unaware; 1 – aware of the links between certain conditions and being overweight.

Age and gender

Control variables were selected on theoretical importance from a rapid review of the literature[21, 40, 43-46] and included gender (coded 0 – Male, 1 – Female) and age (11-19 years).

Patient and Public Involvement

Prior to the development of the survey qualitative research was carried out by colleagues at the University of Stirling and National Centre for Social Research. This work consulted young people through focus groups, on the design and design and content of the questionnaire. Results of these focus group discussions were published in a Cancer Research UK report *"It's Just There To Trick You"*[47]. This PPI development work involved discussion of relevant research questions related to food and beverage consumption, relevant policy issues (i.e. exposure to food marketing, pricing, availability), use of broadcast media examples and the appropriateness of questions relating to socio-demographic characteristics. Questions included in the resulting survey were then trialled with young people using cognitive interviewing techniques as described above. Survey reports are publicly available on Cancer Policy Research Centre's website.

ETHICS

Ethical approval was obtained in January 2017 from the General University Ethics Panel (GUEP) at the University of Stirling (GUEP59). This ethical approval covered both cognitive testing of the questionnaires and the online surveys. YouGov's staff included lead for ethical and quality assurance, to ensure adherence to best practice throughout testing and data collection. This included ensuring informed consent was obtained from participants, links were provided to relevant support organisations should participants wish to contact them and confidentiality of personal information was assured.

ANALYSIS

Data were analysed using IBM SPSS Version 23. Multiple multivariate binary regression models were run on the unweighted data to test for associations between deprivation levels and three key behaviours of young people; consumption behaviours, screen time use and health knowledge. The consumption model used the dependent binary variables of food and drink consumption behaviours. Models were run separately for each dependent variable, producing 15 models in total. The screen time model used the dependent variable of categorised reported screen time hours. The health knowledge model used the dependent binary variable of awareness of a health condition and its link to

For peer review only

overweight and obesity. This included eight different health conditions, some with identified associations, and some without.

Within each of these models the Index of Multiple Deprivation (IMD) variable was used as an independent variable, with the least deprived quintile as the reference group. Age and gender were included in the models as control variables, as potentially confounding variables.

RESULTS

Sample characteristics

Almost half (49%) of the survey respondents were female and 51% male. The mean age of participants was 14.9 years old (SD = 2.55). The majority (82%) were from white British backgrounds with 18% from other ethnic groups. The majority of respondents lived in England (82%); 5% of respondents lived in Wales, 8% in Scotland and 3% in Northern Ireland (*Table 1*).

Screen time

Of respondents, there were 31.9% in low, 57.1% in medium and 11.0% in high screen time category for television viewing. For streaming services there was 30.3% in low, 50.7% in medium and 19.0% in high screen time categories.

Deprivation and Consumption Behaviours

The results of the binary logistic regressions showed an association between deprivation and higher consumption behaviours, for a range of HFSS food products (*Table 2*). The most deprived young people were significantly more likely to consume energy drinks (OR= 2.943, P < 0.001), followed by sugary drinks (OR= 1.938, P < 0.001).

In contrast, analysis identified consumption of fruit and vegetables was inversely associated with more deprived groups. Fruit (OR= 0.668, P =0.004) and vegetables (OR= 0.306, P < 0.001) were more likely to be consumed in lower frequency by the most deprived respondents, when compared to the most affluent respondents, as per the use of IMD. Therefore, these young people had a reduced likelihood of consuming the healthier options in higher quantities.

Deprivation and Screen Time

Regression analysis found an association between deprivation in young people and high weekly screen time of both television and streaming (*Table 3*). The model compared 'high' category screen time (21 hours or more a week) to 'medium and low' screen time (less than 21 hours a week) and found those from the most deprived quintile were significantly more likely to be in the high screen time category than the more affluent respondents, for both television (OR= 2.477, P < 0.001) and streaming (OR= 1.679, P = 0.001).

Deprivation and Health Knowledge

The analysis identified an association between deprivation and poorer health knowledge (*Table 4*). Respondents were asked whether eight health conditions (from a pre-existing list) could occur as a result of being overweight or obese. There was significantly poorer awareness of the association between cancer (OR= 0.697, P = 0.003), type 2 diabetes (OR= 0.64, P = 0.004) and heart disease (OR= 0.519, P < 0.001) and obesity for those from the more deprived quintiles. There was also significantly higher association between incorrectly linking type 1diabetes (OR= 1.536, P < 0.001) and obesity in the most deprived quintile, compared to the most affluent quintile.

For peer review only

DISCUSSION

Results from this survey identify a clear association between socio-economic deprivation and risk factors that may influence the prevalence of childhood obesity in the UK. Involving a nationally representative sample of young people aged 11-19 across England and each of the devolved nations, it sought to explore whether young people living in more deprived areas reported knowledge and behaviours that may contribute to obesity. The study found that these young people consumed more foods and beverages high in salt sugar and fat, and were conversely less likely to report consumption of fruit and vegetables. In addition, young people living in more deprived communities spent more time watching commercial broadcast media where they could be exposed to HFSS advertising. Young people living in less affluent areas also had lower levels of awareness of the preventable health conditions, including cancer, which can arise as a result of obesity.

These results support findings from previous studies on factors influencing childhood obesity but also provide new evidence on the clustering of these factors amongst less affluent groups. It is well established that there is a clear gradient in overweight and obesity by socio-economic status in both adults and young people, with individuals from less affluent communities more likely to carry excess weight compared to their more affluent neighbours[2, 25, 26, 48-50]. More limited research has explored how eating patterns vary by deprivation in young people. This study adds to existing evidence suggesting that greater HFSS consumption and lower levels of fruit and vegetable consumption are more common in less affluent young people[18, 51].

This survey also asked young people about the time they spend watching television and on-demand screening services and calculated ‘screen time’ using an approach employed in previous studies[17, 21]. While higher levels of screen time are associated with sedentary behaviour, which may contribute to obesity, they may also suggest greater exposure to broadcast media marketing including of HFSS foods. Previous research has found that children who spent more time watching commercial TV and on demand programmes in the UK are exposed to more HFSS food marketing than those with lower levels of screen time[24, 52]. Viewing more HFSS ads on TV and streaming has been associated with higher HFSS consumption, with the difference between a high consumer and a low consumer being at least 520 junk food products a year[17].

We also found that young people living in more deprived areas had lower levels of awareness of the links between overweight and obesity and relevant health conditions. Awareness is relevant because evidence relating to other preventable risk factors (such as smoking and alcohol) suggest that health knowledge is relevant as a preliminary step towards changing behaviour, but also, importantly, understanding and support for policies and interventions that may address key factors that drive consumption including restrictions on marketing and pricing of unhealthy products[35, 36, 53, 54].

Taken together our findings suggest that inequalities in rates of obesity in young people in the UK may be linked to knowledge and behaviours driven by key aspects of an obesogenic environment. Action to address childhood obesity needs to take into account differential consumption patterns amongst less affluent young people and the factors that may influence these consumption patterns. The introduction of policies and interventions that aim to address these factors, including better information on the health consequences of obesity, reducing exposure to HFSS marketing and other wider population level measures (such as policies to address the price and content of products) should consider and assess their impact on less affluent groups.

Strengths and limitations of the study

This study has a number of limitations. Data are from a single cross-sectional survey, so it is not possible to determine causation. The measure of deprivation used was an areas-based measure (IMD) which has limitations, as individual or household deprivation may vary within areas. Young people may have limited knowledge and awareness of different health conditions, for example the distinction

For peer review only

between Type 1 and Type II diabetes and therefore responses to the question relating to health conditions may in part reflect this lack of understanding. Responses to each of the key topics of interest including food consumption patterns, use of streaming services and TV viewing, and awareness of health conditions linked to obesity were based on self-report and thus subject to mis-reporting or recall bias. The study could not directly assess exposure to HFSS marketing, although our previous research has explored the relationship between commercial screen time and exposure and found the two to be related[17, 21], which provides us with greater confidence that screen time may be a reliable proxy for marketing exposure under current UK marketing regulations. The response rate for this survey is estimated at 26% for 16-19 year olds and 47% for 11-15 year olds with the parental consent process. This is in line with what is usually obtained for surveys with young people by YouGov and is a limitation when sampling from this population demographic. Finally, overweight and obesity in young people is driven by a wide range of factors beyond those assessed in this study.

Conclusions and future research

Taken together our findings suggest that inequalities in rates of obesity in young people in the UK may be linked to knowledge and behaviours driven by key aspects of an obesogenic environment. Action to address childhood obesity needs to take into account differential consumption patterns amongst less affluent young people and the factors that may influence these consumption patterns. The introduction of policies and interventions that aim to address these factors, including better information on the health consequences of obesity, reducing exposure to HFSS marketing and other wider population level measures (such as policies to address the price and content of products) should consider and assess their impact on less affluent groups.

Future research should explore in more detail a larger number of factors, including, for example, the affordability and availability of HFSS foods, social norms and the influence of social networks in more deprived communities and how these influence knowledge and behaviour among more deprived young people. In addition, studies should assess the impact of changes to the policy and regulatory environment proposed in the UK and other counties to reduce childhood obesity and how these changes may affect young people living in communities where obesity rates are highest.

FUNDING AND ACKNOWLEDGEMENTS

We are grateful to the Cancer Policy Research Centre, Cancer Research UK for funding the study. The views expressed are those of the researchers and not necessarily those of the funder.

CONTRIBUTORSHIP STATEMENT

Fiona Thomas carried out the majority of the analysis for this publication along with Chris Thomas and Lucie Hooper who also developed the questions for the survey. Gillian Rosenberg contributed to the preparation of the manuscript and analysis plan. Both Jyotsna Vohra and Linda Bauld contributed to the study design and concept and the preparation of the manuscript.

COMPETING INTERESTS

None

DATA SHARING AGREEMENT

Data can be made available on request to the Cancer Policy Research Centre (CRUK). Please contact CPRC@cancer.org.uk

For peer review only

Table 1: Sample Demographics of the UK Representative respondents

Male	11 to 12	11.0%
	13 to 15	16.0%
	16 to 17	12.0%
	18 to 19	12.0%
Female	11 to 12	10.0%
	13 to 15	16.0%
	16 to 17	11.0%
	18 to 19	12.0%
Ethnicity	White	82.0%
	BME	18.0%
IMD	1,2	20.0%
	3,4	20.0%
	5,6	20.0%
	7,8	20.0%
	9,10	20.0%
Region	North East	4.0%
	North West	11.1%
	Yorkshire & Humber	8.5%
	East Midlands	7.3%
	West Midlands	9.3%
	East	9.3%
	London	12.7%
	South East	14.0%
	South West	8.2%
	Wales	4.7%
	Scotland	7.8%
	Northern Ireland	3.1%

For peer review only

Table 2: Consumption behaviours and deprivation. The bolded values indicate significance from the model.

	Consumption Behaviours						
	Descriptive Findings				Logistic Regression / Significance		
	Most Deprived 20%		Least Deprived 20%		Most deprived quintile		
	High Consumption (%)	Low Consumption (%)	High Consumption (%)	Low Consumption (%)	OR:	CI (95%)	P - value
**Biscuits and cakes**	58.4%	41.6%	63.6%	36.4%	0.841	0.638 - 1.038	0.097
**Chips**	72.1%	27.9%	67.5%	32.5%	1.259	0.974 - 1.627	0.079
**Confectionary**	65.3%	34.7%	66.3%	33.7%	0.962	0.751 - 1.232	0.759
**Crisps**	62.8%	37.2%	58.5%	41.5%	1.232	0.965 - 1.572	0.093
**Desserts**	47.1%	52.9%	55.2%	44.8%	**0.732**	**0.576 - 0.930**	**0.011**
**Diet Drinks**	35.7%	64.3%	31.2%	68.8%	1.233	0.959 - 1.585	0.102
**Energy drinks**	15.6%	84.4%	7.0%	93.0%	**2.493**	**1.676 - 3.706**	**0.000**
**Flavoured yogurts**	28.0%	72.0%	27.3%	72.7%	1.061	0.812 - 1.386	0.067
**Fruit**	71.3%	28.7%	78.8%	21.2%	**0.668**	**0.507 - 0.879**	**0.004**
**Milk based drinks**	31.3%	68.7%	22.6%	77.4%	**1.613**	**1.229 - 2.118**	**0.001**
**Ready meals**	64.3%	35.7%	56.3%	43.7%	**1.416**	**1.111 - 1.712**	**0.005**
**Sugary drinks**	41.6%	58.4%	27.2%	72.8%	**1.938**	**1.506 - 2.494**	**0.000**
**Sweetened cereals**	49.6%	50.4%	44.8%	55.2%	1.253	0.986 - 1.593	0.066
**Take-aways**	39.1%	60.9%	25.1%	74.9%	**1.914**	**1.482 - 2.472**	**0.000**
**Vegetables**	78.9%	21.1%	92.4%	7.6%	**0.306**	**0.211 - 0.442**	**0.000**

For peer review only

Table 3: Screen-time behaviour and deprivation. The bolded values indicate significance from the model.

	**Screen Time Behaviours**								
	**Frequency**						**Logistic Regression / Significance**		
	**Most Deprived (20%)**			**Least Deprived (20%)**			**Most Deprived Quintile**		
	Low Viewing %	Medium Viewing %	High Viewing %	Low Viewing %	Medium Viewing %	High Viewing %	OR	CI	P-Value
**Television Screen Time**	25.7%	55.9%	18.4%	31.7%	60.0%	8.3%	**2.477**	**1.697 - 3.614**	**0.000**
**Streaming Screen Time**	28.2%	46.8%	25.0%	33.2%	50.3%	16.5%	**1.679**	**1.234 - 2.283**	**0.001**

For peer review only

Table 4: Health knowledge and deprivation. The bolded values indicate significance from the model.

	Overall awareness	Frequency		Logistic Regression / Significance	
		Most Deprived Awareness %	Least Deprived Awareness %	OR	P Value
**Cancer Link**	42.0%	36.1%	44.4%	**0.697**	**[0.003]**
**Heart Disease Link**	87.0%	83.5%	90.3%	**0.519**	**[0.000]**
**Stroke Link**	60.0%	62.1%	59.2%	0.862	[0.228]
**Diabetes Type 1 Link**	39.0%	46.1%	36.3%	**1.536**	**[0.000]**
**Diabetes Type 2 Link**	82.0%	78.7%	84.7%	**0.64**	**[0.004]**
**Flu Link**	4.0%	4.1%	2.7%	1.544	[0.196]
**Chicken Pox Link**	1.0%	0.4%	0.7%	0.558	[0.502]
**Migraine Link**	14.0%	11.0%	11.8%	0.907	[0.604]

For peer review only

References:

1. National Child Measurement Programme (NCMP): Trends in child BMI. Public Health England; 2017.
2. Patterns and Trends in Child Obesity. 2018. https://www.gov.uk/guidance/phe-data-and-analysis-tools#obesity-diet-and-physical-activity2018).
3. Simmonds M, Llewellyn A, Owen CG, Woolacott N. Predicting adult obesity from childhood obesity: a systematic review and meta-analysis. *Obes Rev* 2016; **17**(2): 95-107.
4. Parkin DM, Boyd, L. 8. Cancers attributable to overweight and obesity in the UK in 2010. *Br Journal of Cancer* 2011; **105 Suppl 2**: S34-7.
5. Ades PA, Savage PD. Obesity in coronary heart disease: An unaddressed behavioural risk factor. *Prev Med* 2017; **104**: 117-9
6. Brown KF, Rumgay H, Dunlop C, et al. The fraction of cancer attributable to modifiable risk factors in England, Wales, Scotland, Northern Ireland, and the United Kingdom in 2015. *Br J Cancer* 2018; **118**(8): 1130-41.
7. Adult obesity and type 2 diabetes. Public Health England; 2014.
8. World Health Organisation. Global Health Risks-Mortality and burden of disease attributable to selected major risks. *The Lancet* 2015.
9. Bleich S, Cutler D, Murray C, Adams A. Why is the developed world obese? *Annu Rev Public Health* 2008; **29**: 273-95
10. Ebbeling CB, Pawlak DB, Ludwig DS. Childhood obesity: public-health crisis, common sense cure. *Lancet* 2002; **360**(9331): 473-82
11. Reilly JJ. Obesity in childhood and adolescence: evidence based clinical and public health perspectives. *Postgrad Med J* 2006; **82**(969): 429-37
12. Afshin A, Penalvo JL, Del Gobbo L, et al. The prospective impact of food pricing on improving dietary consumption: A systematic review and meta-analysis. *PLoS One* 2017; **12**(3): e0172277.
13. Vandevijvere S, Chow CC, Hall KD, Umali E, Swinburn BA. Increased food energy supply as a major driver of the obesity epidemic: a global analysis. *Bull World Health Organ* 2015; **93**(7): 446-56
14. Obesity Health Alliance. Health costs of obesity soaring as junk food companies pour millions into advertising. 2017
15. Powell L, Harris J, Fox T. Food Marketing Expenditures Aimed at Youth Putting the Numbers in Context. *American Journal of Preventative Medicine* 2014; **45**(4): 453-61.
16. Story M, French S. Food Advertising and Marketing Directed at Children and Adolescents in the US. *Int J Behav Nutr Phys Act* 2004; **1**(1): 3.
17. Thomas C, Hooper L, Petty R, Thomas F, Rosenberg G, Vohra J. 10 Years On: Cancer Research UK, 2018.
18. Thomas C, Hooper L, Petty R, Thomas F, Rosenberg G, Vohra J. Under Pressure: New evidence on young people's broadcast marketing exposure in the UK: Cancer Research UK, 2018.
19. Thomas F, Hooper L, Petty R, Thomas C, Rosenberg G, Vohra J. A Prime Time for Action: New evidence on the link between television and on-demand marketing and obesity: Cancer Research UK, 2018.
20. Boyland EJ, Nolan S, Kelly B, et al. Advertising as a cue to consume: a systematic review and meta-analysis of the effects of acute exposure to unhealthy food and nonalcoholic beverage advertising on intake in children and adults. *Am J Clin Nutr* 2016; **103**(2): 519-33.

For peer review only

21. Scully M, Wakefield M, Niven P, et al. Association between food marketing exposure and adolescents' food choices and eating behaviors. *Appetite* 2012; **58**(1): 1-5
22. Halford JC, Boyland EJ, Hughes G, Oliveira LP, Dovey TM. Beyond-brand effect of television (TV) food advertisements/commercials on caloric intake and food choice of 5-7-year-old children. *Appetite* 2007; **49**(1): 263-7
23. National Child Measurement Programme. Changes in children's body mass index between 2006/07 and 2015/16.: Public Health England, 2017
24. Boyland EJ, Harrold JA, Kirkham TC, Halford JC. The extent of food advertising to children on UK television in 2008. *International Journal of Pediatric Obesity* 2011; **6**(5-6): 455-61.
25. Wang Y. Cross-national comparison of childhood obesity: the epidemic and the relationship between obesity and socioeconomic status. *Int J Epidemiol* 2001; **30**(5): 1129-36.
26. Public Health England. Severe obesity in ten to eleven year olds reaches record high. In: Mills J, editor.: Public Health England; 2018
27. Wilsher, S. H., Harrison, F., Yamoah, F., Fearne, A., & Jones, A. (2016). The relationship between unhealthy food sales, socio-economic deprivation and childhood weight status: results of a cross-sectional study in England. *International Journal of Behavioral Nutrition and Physical Activity*, *13*(1), 21.
28. Sanderson, S. C., Waller, J., Jarvis, M. J., Humphries, S. E., & Wardle, J. (2009). Awareness of lifestyle risk factors for cancer and heart disease among adults in the UK. *Patient education and counseling*, *74*(2), 221-227.
29. Puhl, R., & Suh, Y. (2015). Health consequences of weight stigma: implications for obesity prevention and treatment. *Current obesity reports*, *4*(2), 182-190.
30. A Healthier Future - Scotland's Diet and Healthy Weight Delivery Plan. Edinburgh: Scottish Government, 2018.
31. Department of Health and Social Care: Global Public Health Directorate: Obesity FaN. Childhood Obesity: a plan for action. Chapter 2: HM Government, 2018
32. Swinburn BA. Obesity prevention: the role of policies, laws and regulations. *Aust New Zealand Health Policy* 2008; **5**: 12
33. WHO. World Health Organisation: Obesity and Overweight. 2017. http://www.who.int/en/news-room/fact-sheets/detail/obesity-and-overweight (accessed 24.07.18 2018).
34. Hooper L, Anderson A, Birch J, et al. Public awareness and healthcare professional advice for obesity as a risk factor for cancer in the UK: a cross-sectional survey. *Journal of Public Health* 2017.
35. Buykx P, Li J, Lovatt M, et al. An Examination of Public Attitudes Towards Alcohol Policy: University of Sheffield and Cancer Research UK, 2016
36. Bates S, Holmes J, Gavens L, et al. Awareness of alcohol as a risk factor for cancer is associated with public support for alcohol policies. *BMC Public Health* 2018; **18**(1): 688
37. Public Health England. National Diet and Nutrition Survey. 2016. https://www.gov.uk/government/collections/national-diet-and-nutrition-survey (accessed 03.01.18 2018)
38. Marketing IoS. Food Choice Survey: University of Stirling, 2007
39. Alcohol (Minimum Unit) Pricing Act 2012. http://www.legislation.gov.uk/asp/2012/4/pdfs/asp_20120004_en.pdf
40. Morley BC, Scully ML, Niven PH, et al. What factors are associated with excess body weight in Australian secondary school students? *Med J Aust* 2012; **196**(3): 189-92.
41. Department for Communities and Local Government. The English Index of Multiple Deprivation (IMD) - Guidance, 2015

42. Sugar Reduction: Achieving the 20%: Public Health England, 2017
43. Dixon HG, Scully ML, Wakefield MA, White VM, Crawford DA. The effects of television advertisements for junk food versus nutritious food on children's food attitudes and preferences. *Soc Sci Med* 2007; **65**(7): 1311-23.
44. Denny S, Lewycka S, Utter J, et al. The association between socioeconomic deprivation and secondary school students' health: findings from a latent class analysis of a national adolescent health survey. *Int J Equity Health* 2016; **15**(1): 109
45. Nestle M. Food marketing and childhood obesity--a matter of policy. *N Engl J Med* 2006; **354**(24): 2527-9.
46. Cooke LJ, Wardle J. Age and gender differences in children's food preferences. *Br J Nutr* 2005; **93**(5): 741-6.
47. MacGregor, A., Bicquelet, A., Lepps. H., Porter, L., Eadie, D., McKell, J., MacKintosh, A A., Thomas, C., Hooper, L and Vohra, J (2016) *""It's just there to trick you": A qualitative study of 11-19 year olds perceptions of food and drink marketing."* ScotCen Social Research; NatCen Social Research; Institute for Social Marketing, University of Stirling; and Policy Research Centre for Cancer Prevention, Cancer Research UK.
48. Stamatakis E, Wardle J, Cole TJ. Childhood obesity and overweight prevalence trends in England: evidence for growing socioeconomic disparities. *Int J Obes (Lond)* 2010; **34**(1): 41-7
49. McLaren L. Socioeconomic status and obesity. *Epidemiol Rev* 2007; **29**: 29-48
50. Bann D, Johnson W, Li L, Kuh D, Hardy R. Socioeconomic Inequalities in Body Mass Index across Adulthood: Coordinated Analyses of Individual Participant Data from Three British Birth Cohort Studies Initiated in 1946, 1958 and 1970. *PLoS Med* 2017; **14**(1): e1002214
51. Crawford B, Byun R, Mitchell E, Thompson S, Jalaludin B, Torvaldsen S. Socioeconomic differences in the cost, availability and quality of healthy food in Sydney. *Aust N Z J Public Health* 2017; **41**(6): 567-71
52. Campbell D. Children seeing up to 12 adverts for junk food an hour on TV, study finds. 2017. https://www.theguardian.com/society/2017/nov/28/children-seeing-up-to-12-adverts-for-junk-food-an-hour-on-tv-study-finds (accessed 23.07.18 2018)
53. Bryant T. Role of knowledge in public health and health promotion policy change. *Health Promot Int* 2002; **17**(1): 89-98
54. Raphael D and Bryant T (2006) The state's role in promoting population health: Public health concerns in Canada, USA, UK and Sweden. *Health Policy.* 78 (1) 39 - 55

For peer review only

STROBE Statement—Checklist of items that should be included in reports of ***cross-sectional studies***

	Item No	Recommendation	Page No.
**Title and abstract**	1	(*a*) Indicate the study's design with a commonly used term in the title or the abstract	1
		(*b*) Provide in the abstract an informative and balanced summary of what was done and what was found	2
**Introduction**			
Background/rationale	2	Explain the scientific background and rationale for the investigation being reported	5
Objectives	3	State specific objectives, including any prespecified hypotheses	5
**Methods**			
Study design	4	Present key elements of study design early in the paper	6
Setting	5	Describe the setting, locations, and relevant dates, including periods of recruitment, exposure, follow-up, and data collection	6
Participants	6	(*a*) Give the eligibility criteria, and the sources and methods of selection of participants	6
Variables	7	Clearly define all outcomes, exposures, predictors, potential confounders, and effect modifiers. Give diagnostic criteria, if applicable	6
Data sources/ measurement	8*	For each variable of interest, give sources of data and details of methods of assessment (measurement). Describe comparability of assessment methods if there is more than one group	6
Bias	9	Describe any efforts to address potential sources of bias	6
Study size	10	Explain how the study size was arrived at	6
Quantitative variables	11	Explain how quantitative variables were handled in the analyses. If applicable, describe which groupings were chosen and why	6, 7
Statistical methods	12	(*a*) Describe all statistical methods, including those used to control for confounding	6, 7
		(*b*) Describe any methods used to examine subgroups and interactions	7
		(*c*) Explain how missing data were addressed	Not recorded for this study as online survey was used
		(*d*) If applicable, describe analytical methods taking account of sampling strategy	6
		(*e*) Describe any sensitivity analyses	NR
**Results**			
Participants	13*	(a) Report numbers of individuals at each stage of study—eg numbers potentially eligible, examined for eligibility, confirmed eligible, included in the study, completing follow-up, and analysed	8

For peer review only

		(b) Give reasons for non-participation at each stage	This information was not collected.
		(c) Consider use of a flow diagram	-
Descriptive data	14*	(a) Give characteristics of study participants (eg demographic, clinical, social) and information on exposures and potential confounders	8, 11
		(b) Indicate number of participants with missing data for each variable of interest	6
Outcome data	15*	Report numbers of outcome events or summary measures	8
Main results	16	(*a*) Give unadjusted estimates and, if applicable, confounder-adjusted estimates and their precision (eg, 95% confidence interval). Make clear which confounders were adjusted for and why they were included	12,13,14
		(*b*) Report category boundaries when continuous variables were categorized	12,13,14
		(*c*) If relevant, consider translating estimates of relative risk into absolute risk for a meaningful time period	NR
Other analyses	17	Report other analyses done—eg analyses of subgroups and interactions, and sensitivity analyses	NR
**Discussion**			
Key results	18	Summarise key results with reference to study objectives	8
Limitations	19	Discuss limitations of the study, taking into account sources of potential bias or imprecision. Discuss both direction and magnitude of any potential bias	9,10
Interpretation	20	Give a cautious overall interpretation of results considering objectives, limitations, multiplicity of analyses, results from similar studies, and other relevant evidence	9,10
Generalisability	21	Discuss the generalisability (external validity) of the study results	9,10
**Other information**			
Funding	22	Give the source of funding and the role of the funders for the present study and, if applicable, for the original study on which the present article is based	10

*Give information separately for exposed and unexposed groups.

Note: An Explanation and Elaboration article discusses each checklist item and gives methodological background and published examples of transparent reporting. The STROBE checklist is best used in conjunction with this article (freely available on the Web sites of PLoS Medicine at http://www.plosmedicine.org/, Annals of Internal Medicine at http://www.annals.org/, and Epidemiology at http://www.epidem.com/). Information on the STROBE Initiative is available at www.strobe-statement.org.

For peer review only - http://bmjopen.bmj.com/site/about/guidelines.xhtml

BMJ Open

Area deprivation, screen time and consumption of food and drink high in fat salt and sugar (HFSS) in young people: Results from a cross sectional study in the UK

Journal:	*BMJ Open*
Manuscript ID	bmjopen-2018-027333.R3
Article Type:	Research
Date Submitted by the Author:	06-Jun-2019

Complete List of Authors:	Thomas, Fiona; Cancer Research UK, Cancer Prevention<br>Thomas, Christopher; Cancer Research UK, Policy Research Centre for Cancer Prevention<br>Hooper, Lucie; Cancer Research UK, Policy Research Centre for Cancer Prevention<br>Rosenberg, Gillian; Cancer Research UK, Policy Research Centre for Cancer Prevention<br>Vohra, Jyotsna; Cancer Research UK, Cancer Prevention<br>Bauld, Linda; University of Edinburgh, Usher Institute of Population Health Sciences and Informatics

**Primary Subject Heading**:	Public health
Secondary Subject Heading:	Nutrition and metabolism, Health policy, Public health
Keywords:	young people, inequalities, consumption, screen time, health knowledge, deprivation

SCHOLARONE™ Manuscripts

Area deprivation, screen time and consumption of food and drink high in fat salt and sugar (HFSS) in young people: Results from a cross sectional study in the UK

Fiona Thomas [1], Christopher Thomas [1], Lucie Hooper [1], Gillian Rosenberg [1], Jyotsna Vohra [1] and Linda Bauld[1, 2]
[1]Cancer Policy Research Centre, Cancer Research UK
[2]Usher Institute, University of Edinburgh

Key words: Obesity, Young people, Inequalities, Consumption, Screen time, Health Knowledge, Deprivation

Correspondence to: Fiona Thomas, Cancer Research UK, Angel Building, 407 St. John Street London, EC1V 4AD

Contact Details:

Fiona Thomas – fiona.thomas@hotmail.com
Christopher Thomas – Christopher.Thomas@cancer.org.uk
Lucie Hooper – Lucie.Hooper@cancer.org.uk
Gillian Rosenberg – Gillian.Rosenberg@cancer.org.uk
Jyotsna Vohra – Jyotsna.Vohra@cancer.org.uk
Linda Bauld – Linda.Bauld@ed.ac.uk

Word Count: 3289

Number of Tables: 4
Number of References: 54

Corresponding Author:

Fiona Thomas

Cancer Policy Research Centre

Cancer Research UK

Angel Building

407 St. John Street

London

EC1V 4AD

United Kingdom

Tel - 020 3469 6357

Email - fiona.thomas@hotmail.com

For peer review only

Abstract

Objectives: To investigate associations between deprivation in young people and consumption of foods high in fat, salt and sugar (HFSS), screen time exposure and health knowledge.

Design: An online cross-sectional survey with 11-19-year olds in the UK, where participants reported consumption behaviours across 13 HFSS and two non-HFSS groups; screen time for commercial television and streaming services; and knowledge of health conditions and their links to obesity.

Setting: United Kingdom

Participants: 3,348 young people aged 11-19 across the United Kingdom (UK).

Main outcome measures: The study assessed the consumption behaviours, commercial screen time exposure and the health knowledge of 3,348 11-19-year olds. Multivariate binary regression analysis, controlling for age and gender, was performed.

Results: Deprivation level was associated with increases in consumption of six of the HFSS products including energy drinks (OR:2.943 / P< 0.001) and sugary drinks (OR:1.938, P< 0.001), and a reduction in consumption in the two non-HFSS products included in the study; fruit (OR:0.668 / P=0.004) and vegetables (OR:0.306 / P< 0.001). Deprivation was associated with high weekly screen time of both television (OR:2.477 / P< 0.001) and streaming (OR:1.679 / P=0.001). Health knowledge was also associated with deprivation. There was lower awareness of the association of obesity and cancer (OR:0.697 / P=0.003) type 2 diabetes (OR:0.64 / P=0.004) and heart disease (OR:0.519 / P< 0.001) in the most deprived.

Conclusions: Young people from the more deprived areas of the UK were more likely to consume a range of HFSS products, report increased exposure to HFSS advertising and have a poorer awareness of health conditions associated with overweight and obesity. The findings suggest that population level measures addressing childhood obesity should account for consumption patterns among different groups of children and young people and the factors that may influence these.

For peer review only

Strengths and limitations of this study

- The study identified an association between socio-economic deprivation and risk factors that may influence the prevalence of childhood obesity in the UK through a nationally representative sample of young people aged 11-19 across England and each of the devolved nations.
- Data collected for this study was from a single cross-sectional survey, so it is not possible to determine causation between the variables.
- The study could not directly assess exposure to marketing of foods high in fat, salt and sugar (HFSS), although previous research that had explored the relationship between commercial screen time and exposure and found the two to be related, provided us with greater confidence that screen time may be a reliable proxy for marketing exposure under current UK marketing regulations.

For peer review only

BACKGROUND

In the UK, around 30% of children are overweight or obese[1] the highest rate of childhood obesity in Europe. This overall figure masks considerable disparities by socioeconomic status. Overweight and obesity prevalence for children in the 10% most deprived areas in England, for example, is more than double that of those who live in the least deprived 10%[2]. Longer term, an obese child is around five times more likely to become an obese adult[3], and there is substantial evidence that obesity in adulthood directly contributes to the development of conditions such as diabetes, coronary heart disease, stroke and 13 different types of cancer[4-8].

Previous studies have provided diverse explanations for the rise in levels of obesity, ranging from genetics, increased calorie intake, an increase in sedentary behaviour or a combination of factors[9-11]. The calorie intake increase is thought to be the most significant influence accounting for this rise, caused by a range of environmental factors[9,12,13], including the role of the marketing and promotion of foods that are high in fat, salt and sugar (HFSS), often referred to as 'junk foods'. Marketing of these foods is extensive and delivered through a variety of platforms including television, streaming, price promotions and print media. Studies have identified a substantial expenditure by manufacturers and retailers on the marketing of junk food to children and young adults[14, 15], and identified that industry recognises the potential marketing has for influencing consumption choices[16]. The link between marketing and weight outcomes, as well as increased consumption of HFSS products has been highlighted by a number of previous studies[17-23]. Assessing exposure to HFSS marketing via self-reported recall of viewing advertisements does have limitations. Thus some studies have used commercial screen time as a proxy for TV and online marketing exposure[17, 21], whereby greater screen time indicates increased exposure to HFSS advertising. Prior content analysis of UK television, where young people make up a large proportion of the audience, highlighted the increased likelihood of HFSS marketing exposure[24], supporting this proxy measure.

Increasingly, there is a need to identify how marketing and promotion affect children in different social groups or those living in more, or less deprived communities. Studies have previously identified an association between socioeconomic status and obesity[25, 26]. This association is likely to be caused by a wide variety of factors including the pricing and availability of particular products in a locality[27], understanding and awareness of dietary factors and weight and social norms[28, 29]. However, further research is required to understand the inter-relationship of these factors and also other drivers of consumption including exposure to HFSS advertising. This type of research is important to identify interventions or policy actions that can contribute to addressing overweight and obesity. Halting or reversing current obesity trends is a current priority for public health policies in the UK[30, 31], and globally[32,33].

Hooper et al[34], identified that there is a low level of public awareness of the link between overweight and obesity and resulting preventable health conditions, including cancer. Only 25% of the UK adult population are aware of this link and this lack of awareness is more prevalent in less affluent groups. Other studies have also found an association between greater health awareness and increased support for policy change, particularly for alcohol policy[35, 36]. Greater health knowledge may therefore affect how young people view the acceptability of HFSS marketing and also consumption choices.

To date, there is limited research on the association between deprivation, HFSS marketing and obesity. Given these gaps, this study aims to investigate whether such a relationship exists and how it might be influenced by particular mediators such as frequency and duration of exposure to marketing and knowledge and understanding of health risks.

For peer review only

METHODS

Study design

An online cross-sectional survey was conducted between April and June 2017. The survey was developed following cognitive testing with a small sample of young people (n=100) to ensure age and cultural comprehensibility of the questions, some of which were based on well validated questions used in other surveys[21, 37-40]. This survey was compiled using the pre-validated questions and the advice of senior researchers from the National Cancer Institute, USA and Public Health England and the comprehensive guidance of the Institute for Social Marketing and the University of Stirling who have experience of running the Youth Tobacco Policy Survey. The final survey covered six main themes; exercise levels; food and drink consumption, screen time, recalled marketing exposure, perceptions of marketing and demographic factors.

A total of 3,348 young people, aged 11-19 were recruited by market research company, YouGov, using their in-house panel. YouGov already had data on the children in households of adult in-house panel members. Children over the age of 16 were directly approached and asked if they wished to participate. For those aged under 16, their parents were contacted and asked if their child could participate in the survey. Data collected was weighted by age, gender, ethnicity, region and social grade to be representative of the UK population.

Measures

Deprivation

Level of deprivation was assessed using an area-based measure rather than individual measure of socio-economic status. The Index of Multiple Deprivation (IMD) was coded into five equal quintiles for analysis, ranging from (1) the lowest 20% of deprivation to (5) the highest 20% of respondents. IMD is a measure of the relative deprivation of an area, combining information from seven domains; income deprivation, employment deprivation, education, skills and training deprivation, health deprivation and disability, crime, barriers to housing and services and living environment deprivation[41].

Consumption Behaviours

The survey measured consumption of a range of food and drink products. Participants were asked to report their consumption behaviours from the question 'How often do you usually eat or drink…?' followed by a series of food categories high in fat, salt and sugar including biscuits and cakes, chips, confectionary, crisps, desserts, energy drinks, flavoured yogurts, milk-based drinks, ready meals, sugary drinks, sweetened cereal and take-aways, as well as with healthy items such as fruit and vegetables. These food groups were chosen using previous research on unhealthy products and with reference to the categories included by Public Health England in their sugar reduction programme[42] and the nutrient profiling model, to ascertain nutritional composition of foods. Responses were graded on a Likert scale from more than once a day to never, and then converted to binary variables across two coding groups. The groups identified as 'higher' consumption depending on the total calorific content in each food[17, 21, 42]. The first coding group included sugar sweetened drinks, flavoured yoghurts, confectionary, cakes/biscuits, fruit, vegetables, diet drinks, crisps and desserts where two or more portions a week was considered high consumption[42]. The second coding group included takeaways, ready meals, energy drinks, fried potato products, milk-based drinks and sugar sweetened cereals where one or more portions a week was considered higher consumption[42]. This coding was deduced from a range of approaches including the Public Health England nutrient model, pilot testing and a review of the average calories in each product, recognising the differences between portion

For peer review only

sizes of the food categories. The coding was only calculated for participants who gave an answer, and those who selected 'not sure' were excluded from the final analysis.

Screen Time

Commercial screen time was a variable created in the data set based on responses related to frequency and duration of exposure to TV and streaming (on demand) services[21, 43]. Participants listed the hours spent watching both commercial and non-commercial TV and streaming services. This excluded screen time from computers being used for homework. Non-commercial screen time (which contains no paid for marketing in the UK context) was shown to not be significant in previous analysis of the data[17-19] and was therefore removed. Weekend and week-day viewing was then weighted and turned into a weekly measure and categorised; low (<3 hours per week), medium (3 – 21 hours per week) and high (21 hours or more per week)[21].

Health Knowledge

Health knowledge was assessed using the question 'Which, if any, of the following health conditions do you think can result from being overweight? Please tick all that apply.' Options included answers that were both correct and incorrect to identify the extent of health knowledge. The eight chosen conditions were; cancer, stroke, heart disease, diabetes type 1, diabetes type 2, migraines, chicken pox and flu. From this, the results were coded as a binary variable: 0 – unaware; 1 – aware of the links between certain conditions and being overweight.

Age and gender

Control variables were selected on theoretical importance from a rapid review of the literature[21, 40, 43-46] and included gender (coded 0 – Male, 1 – Female) and age (11-19 years).

Patient and Public Involvement

Prior to the development of the survey qualitative research was carried out by colleagues at the University of Stirling and National Centre for Social Research. This work consulted young people through focus groups, on the design and design and content of the questionnaire. Results of these focus group discussions were published in a Cancer Research UK report *"It's Just There To Trick You"*[47]. This PPI development work involved discussion of relevant research questions related to food and beverage consumption, relevant policy issues (i.e. exposure to food marketing, pricing, availability), use of broadcast media examples and the appropriateness of questions relating to socio-demographic characteristics. Questions included in the resulting survey were then trialled with young people using cognitive interviewing techniques as described above. Survey reports are publicly available on Cancer Policy Research Centre's website.

ETHICS

Ethical approval was obtained in January 2017 from the General University Ethics Panel (GUEP) at the University of Stirling. This ethical approval covered both cognitive testing of the questionnaires and the online surveys. YouGov's staff included lead for ethical and quality assurance, to ensure adherence to best practice throughout testing and data collection. This included ensuring informed consent was obtained from participants, links were provided to relevant support organisations should participants wish to contact them and confidentiality of personal information was assured.

ANALYSIS

Data were analysed using IBM SPSS Version 23. Multiple multivariate binary regression models were run on the unweighted data to test for associations between deprivation levels and three key behaviours of young people; consumption behaviours, screen time use and health knowledge. The

For peer review only

consumption model used the dependent binary variables of food and drink consumption behaviours. Models were run separately for each dependent variable, producing 15 models in total. The screen time model used the dependent variable of categorised reported screen time hours. The health knowledge model used the dependent binary variable of awareness of a health condition and its link to overweight and obesity. This included eight different health conditions, some with identified associations, and some without.

Within each of these models the Index of Multiple Deprivation (IMD) variable was used as an independent variable, with the least deprived quintile as the reference group. Age and gender were included in the models as control variables, as potentially confounding variables.

RESULTS

Sample characteristics

Almost half (49%) of the survey respondents were female and 51% male. The mean age of participants was 14.9 years old (SD = 2.55). The majority (82%) were from white British backgrounds with 18% from other ethnic groups. The majority of respondents lived in England (82%); 5% of respondents lived in Wales, 8% in Scotland and 3% in Northern Ireland (*Table 1*).

Screen time

Of respondents, there were 31.9% in low, 57.1% in medium and 11.0% in high screen time category for television viewing. For streaming services there was 30.3% in low, 50.7% in medium and 19.0% in high screen time categories.

Deprivation and Consumption Behaviours

The results of the binary logistic regressions showed an association between deprivation and higher consumption behaviours, for a range of HFSS food products (*Table 2*). The most deprived young people were significantly more likely to consume energy drinks (OR= 2.943, $P < 0.001$), followed by sugary drinks (OR= 1.938, $P < 0.001$).

In contrast, analysis identified consumption of fruit and vegetables was inversely associated with more deprived groups. Fruit (OR= 0.668, P =0.004) and vegetables (OR= 0.306, $P < 0.001$) were more likely to be consumed in lower frequency by the most deprived respondents, when compared to the most affluent respondents, as per the use of IMD. Therefore, these young people had a reduced likelihood of consuming the healthier options in higher quantities.

Deprivation and Screen Time

Regression analysis found an association between deprivation in young people and high weekly screen time of both television and streaming (*Table 3*). The model compared 'high' category screen time (21 hours or more a week) to 'medium and low' screen time (less than 21 hours a week) and found those from the most deprived quintile were significantly more likely to be in the high screen time category than the more affluent respondents, for both television (OR= 2.477, $P < 0.001$) and streaming (OR= 1.679, P = 0.001).

Deprivation and Health Knowledge

The analysis identified an association between deprivation and poorer health knowledge (*Table 4*). Respondents were asked whether eight health conditions (from a pre-existing list) could occur as a result of being overweight or obese. There was significantly poorer awareness of the association between cancer (OR= 0.697, P = 0.003), type 2 diabetes (OR= 0.64, P = 0.004) and heart disease (OR= 0.519, $P < 0.001$) and obesity for those from the more deprived quintiles. There was also

For peer review only

significantly higher association between incorrectly linking type 1diabetes (OR= 1.536, $P < 0.001$) and obesity in the most deprived quintile, compared to the most affluent quintile.

For peer review only

DISCUSSION

Results from this survey identify a clear association between socio-economic deprivation and risk factors that may influence the prevalence of childhood obesity in the UK. Involving a nationally representative sample of young people aged 11-19 across England and each of the devolved nations, it sought to explore whether young people living in more deprived areas reported knowledge and behaviours that may contribute to obesity. The study found that these young people consumed more foods and beverages high in salt sugar and fat, and were conversely less likely to report consumption of fruit and vegetables. In addition, young people living in more deprived communities spent more time watching commercial broadcast media where they could be exposed to HFSS advertising. Young people living in less affluent areas also had lower levels of awareness of the preventable health conditions, including cancer, which can arise as a result of obesity.

These results support findings from previous studies on factors influencing childhood obesity but also provide new evidence on the clustering of these factors amongst less affluent groups. It is well established that there is a clear gradient in overweight and obesity by socio-economic status in both adults and young people, with individuals from less affluent communities more likely to carry excess weight compared to their more affluent neighbours[2, 25, 26, 48-50]. More limited research has explored how eating patterns vary by deprivation in young people. This study adds to existing evidence suggesting that greater HFSS consumption and lower levels of fruit and vegetable consumption are more common in less affluent young people[18, 51].

This survey also asked young people about the time they spend watching television and on-demand screening services and calculated 'screen time' using an approach employed in previous studies[17, 21]. While higher levels of screen time are associated with sedentary behaviour, which may contribute to obesity, they may also suggest greater exposure to broadcast media marketing including of HFSS foods. Previous research has found that children who spent more time watching commercial TV and on demand programmes in the UK are exposed to more HFSS food marketing than those with lower levels of screen time[24, 52]. Viewing more HFSS ads on TV and streaming has been associated with higher HFSS consumption, with the difference between a high consumer and a low consumer being at least 520 junk food products a year[17].

We also found that young people living in more deprived areas had lower levels of awareness of the links between overweight and obesity and relevant health conditions. Awareness is relevant because evidence relating to other preventable risk factors (such as smoking and alcohol) suggest that health knowledge is relevant as a preliminary step towards changing behaviour, but also, importantly, understanding and support for policies and interventions that may address key factors that drive consumption including restrictions on marketing and pricing of unhealthy products[35, 36, 53, 54].

Taken together our findings suggest that inequalities in rates of obesity in young people in the UK may be linked to knowledge and behaviours driven by key aspects of an obesogenic environment. Action to address childhood obesity needs to take into account differential consumption patterns amongst less affluent young people and the factors that may influence these consumption patterns. The introduction of policies and interventions that aim to address these factors, including better information on the health consequences of obesity, reducing exposure to HFSS marketing and other wider population level measures (such as policies to address the price and content of products) should consider and assess their impact on less affluent groups.

Strengths and limitations of the study

This study has a number of limitations. Data are from a single cross-sectional survey, so it is not possible to determine causation. The measure of deprivation used was an areas-based measure (IMD) which has limitations, as individual or household deprivation may vary within areas. Young people may have limited knowledge and awareness of different health conditions, for example the distinction

For peer review only

between Type 1 and Type II diabetes and therefore responses to the question relating to health conditions may in part reflect this lack of understanding. Responses to each of the key topics of interest including food consumption patterns, use of streaming services and TV viewing, and awareness of health conditions linked to obesity were based on self-report and thus subject to mis-reporting or recall bias. The study could not directly assess exposure to HFSS marketing, although our previous research has explored the relationship between commercial screen time and exposure and found the two to be related[17, 21], which provides us with greater confidence that screen time may be a reliable proxy for marketing exposure under current UK marketing regulations. The response rate for this survey is estimated at 26% for 16-19 year olds and 47% for 11-15 year olds with the parental consent process. This is in line with what is usually obtained for surveys with young people by YouGov and is a limitation when sampling from this population demographic. Finally, overweight and obesity in young people is driven by a wide range of factors beyond those assessed in this study.

Conclusions and future research

Taken together our findings suggest that inequalities in rates of obesity in young people in the UK may be linked to knowledge and behaviours driven by key aspects of an obesogenic environment. Action to address childhood obesity needs to take into account differential consumption patterns amongst less affluent young people and the factors that may influence these consumption patterns. The introduction of policies and interventions that aim to address these factors, including better information on the health consequences of obesity, reducing exposure to HFSS marketing and other wider population level measures (such as policies to address the price and content of products) should consider and assess their impact on less affluent groups.

Future research should explore in more detail a larger number of factors, including, for example, the affordability and availability of HFSS foods, social norms and the influence of social networks in more deprived communities and how these influence knowledge and behaviour among more deprived young people. In addition, studies should assess the impact of changes to the policy and regulatory environment proposed in the UK and other counties to reduce childhood obesity and how these changes may affect young people living in communities where obesity rates are highest.

FUNDING AND ACKNOWLEDGEMENTS

We are grateful to the Cancer Policy Research Centre, Cancer Research UK for funding the study. The views expressed are those of the researchers and not necessarily those of the funder.

CONTRIBUTORSHIP STATEMENT

Fiona Thomas carried out the majority of the analysis for this publication along with Chris Thomas and Lucie Hooper who also developed the questions for the survey. Gillian Rosenberg contributed to the preparation of the manuscript and analysis plan. Both Jyotsna Vohra and Linda Bauld contributed to the study design and concept and the preparation of the manuscript.

COMPETING INTERESTS

None

DATA SHARING AGREEMENT

Data can be made available on request to the Cancer Policy Research Centre (CRUK). Please contact CPRC@cancer.org.uk

For peer review only

Table 1: Sample Demographics of the UK Representative respondents

Male	11 to 12	11.0%
	13 to 15	16.0%
	16 to 17	12.0%
	18 to 19	12.0%
Female	11 to 12	10.0%
	13 to 15	16.0%
	16 to 17	11.0%
	18 to 19	12.0%
Ethnicity	White	82.0%
	BME	18.0%
IMD	1,2	20.0%
	3,4	20.0%
	5,6	20.0%
	7,8	20.0%
	9,10	20.0%
Region	North East	4.0%
	North West	11.1%
	Yorkshire & Humber	8.5%
	East Midlands	7.3%
	West Midlands	9.3%
	East	9.3%
	London	12.7%
	South East	14.0%
	South West	8.2%
	Wales	4.7%
	Scotland	7.8%
	Northern Ireland	3.1%

For peer review only

Table 2: Consumption behaviours and deprivation. The bolded values indicate significance from the model.

	Consumption Behaviours						
	Descriptive Findings				Logistic Regression / Significance		
	Most Deprived 20%		Least Deprived 20%		Most deprived quintile		
	High Consumption (%)	Low Consumption (%)	High Consumption (%)	Low Consumption (%)	OR:	CI (95%)	P - value
**Biscuits and cakes**	58.4%	41.6%	63.6%	36.4%	0.841	0.638 - 1.038	0.097
**Chips**	72.1%	27.9%	67.5%	32.5%	1.259	0.974 - 1.627	0.079
**Confectionary**	65.3%	34.7%	66.3%	33.7%	0.962	0.751 - 1.232	0.759
**Crisps**	62.8%	37.2%	58.5%	41.5%	1.232	0.965 - 1.572	0.093
**Desserts**	47.1%	52.9%	55.2%	44.8%	**0.732**	**0.576 - 0.930**	**0.011**
**Diet Drinks**	35.7%	64.3%	31.2%	68.8%	1.233	0.959 - 1.585	0.102
**Energy drinks**	15.6%	84.4%	7.0%	93.0%	**2.493**	**1.676 - 3.706**	**0.000**
**Flavoured yogurts**	28.0%	72.0%	27.3%	72.7%	1.061	0.812 - 1.386	0.067
**Fruit**	71.3%	28.7%	78.8%	21.2%	**0.668**	**0.507 - 0.879**	**0.004**
**Milk based drinks**	31.3%	68.7%	22.6%	77.4%	**1.613**	**1.229 - 2.118**	**0.001**
**Ready meals**	64.3%	35.7%	56.3%	43.7%	**1.416**	**1.111 - 1.712**	**0.005**
**Sugary drinks**	41.6%	58.4%	27.2%	72.8%	**1.938**	**1.506 - 2.494**	**0.000**
**Sweetened cereals**	49.6%	50.4%	44.8%	55.2%	1.253	0.986 - 1.593	0.066
**Take-aways**	39.1%	60.9%	25.1%	74.9%	**1.914**	**1.482 - 2.472**	**0.000**
**Vegetables**	78.9%	21.1%	92.4%	7.6%	**0.306**	**0.211 - 0.442**	**0.000**

For peer review only

Table 3: Screen-time behaviour and deprivation. The bolded values indicate significance from the model.

	**Screen Time Behaviours**								
	**Frequency**						**Logistic Regression / Significance**		
	**Most Deprived (20%)**			**Least Deprived (20%)**			**Most Deprived Quintile**		
	Low Viewing %	Medium Viewing %	High Viewing %	Low Viewing %	Medium Viewing %	High Viewing %	OR	CI	P-Value
**Television Screen Time**	25.7%	55.9%	18.4%	31.7%	60.0%	8.3%	**2.477**	**1.697 - 3.614**	**0.000**
**Streaming Screen Time**	28.2%	46.8%	25.0%	33.2%	50.3%	16.5%	**1.679**	**1.234 - 2.283**	**0.001**

For peer review only

Table 4: Health knowledge and deprivation. The bolded values indicate significance from the model.

	Overall awareness	Frequency		Logistic Regression / Significance	
		Most Deprived Awareness %	Least Deprived Awareness %	OR	P Value
**Cancer Link**	42.0%	36.1%	44.4%	**0.697**	**[0.003]**
**Heart Disease Link**	87.0%	83.5%	90.3%	**0.519**	**[0.000]**
**Stroke Link**	60.0%	62.1%	59.2%	0.862	[0.228]
**Diabetes Type 1 Link**	39.0%	46.1%	36.3%	**1.536**	**[0.000]**
**Diabetes Type 2 Link**	82.0%	78.7%	84.7%	**0.64**	**[0.004]**
**Flu Link**	4.0%	4.1%	2.7%	1.544	[0.196]
**Chicken Pox Link**	1.0%	0.4%	0.7%	0.558	[0.502]
**Migraine Link**	14.0%	11.0%	11.8%	0.907	[0.604]

For peer review only

References:

1. National Child Measurement Programme (NCMP): Trends in child BMI. Public Health England; 2017.
2. Patterns and Trends in Child Obesity. 2018. https://www.gov.uk/guidance/phe-data-and-analysis-tools#obesity-diet-and-physical-activity2018).
3. Simmonds M, Llewellyn A, Owen CG, Woolacott N. Predicting adult obesity from childhood obesity: a systematic review and meta-analysis. *Obes Rev* 2016; **17**(2): 95-107.
4. Parkin DM, Boyd, L. 8. Cancers attributable to overweight and obesity in the UK in 2010. *Br Journal of Cancer* 2011; **105 Suppl 2**: S34-7.
5. Ades PA, Savage PD. Obesity in coronary heart disease: An unaddressed behavioural risk factor. *Prev Med* 2017; **104**: 117-9
6. Brown KF, Rumgay H, Dunlop C, et al. The fraction of cancer attributable to modifiable risk factors in England, Wales, Scotland, Northern Ireland, and the United Kingdom in 2015. *Br J Cancer* 2018; **118**(8): 1130-41.
7. Adult obesity and type 2 diabetes. Public Health England; 2014.
8. World Health Organisation. Global Health Risks-Mortality and burden of disease attributable to selected major risks. *The Lancet* 2015.
9. Bleich S, Cutler D, Murray C, Adams A. Why is the developed world obese? *Annu Rev Public Health* 2008; **29**: 273-95
10. Ebbeling CB, Pawlak DB, Ludwig DS. Childhood obesity: public-health crisis, common sense cure. *Lancet* 2002; **360**(9331): 473-82
11. Reilly JJ. Obesity in childhood and adolescence: evidence based clinical and public health perspectives. *Postgrad Med J* 2006; **82**(969): 429-37
12. Afshin A, Penalvo JL, Del Gobbo L, et al. The prospective impact of food pricing on improving dietary consumption: A systematic review and meta-analysis. *PLoS One* 2017; **12**(3): e0172277.
13. Vandevijvere S, Chow CC, Hall KD, Umali E, Swinburn BA. Increased food energy supply as a major driver of the obesity epidemic: a global analysis. *Bull World Health Organ* 2015; **93**(7): 446-56
14. Obesity Health Alliance. Health costs of obesity soaring as junk food companies pour millions into advertising. 2017
15. Powell L, Harris J, Fox T. Food Marketing Expenditures Aimed at Youth Putting the Numbers in Context. *American Journal of Preventative Medicine* 2014; **45**(4): 453-61.
16. Story M, French S. Food Advertising and Marketing Directed at Children and Adolescents in the US. *Int J Behav Nutr Phys Act* 2004; **1**(1): 3.
17. Thomas C, Hooper L, Petty R, Thomas F, Rosenberg G, Vohra J. 10 Years On: Cancer Research UK, 2018.
18. Thomas C, Hooper L, Petty R, Thomas F, Rosenberg G, Vohra J. Under Pressure: New evidence on young people's broadcast marketing exposure in the UK: Cancer Research UK, 2018.
19. Thomas F, Hooper L, Petty R, Thomas C, Rosenberg G, Vohra J. A Prime Time for Action: New evidence on the link between television and on-demand marketing and obesity: Cancer Research UK, 2018.
20. Boyland EJ, Nolan S, Kelly B, et al. Advertising as a cue to consume: a systematic review and meta-analysis of the effects of acute exposure to unhealthy food and nonalcoholic beverage advertising on intake in children and adults. *Am J Clin Nutr* 2016; **103**(2): 519-33.

For peer review only

21. Scully M, Wakefield M, Niven P, et al. Association between food marketing exposure and adolescents' food choices and eating behaviors. *Appetite* 2012; **58**(1): 1-5
22. Halford JC, Boyland EJ, Hughes G, Oliveira LP, Dovey TM. Beyond-brand effect of television (TV) food advertisements/commercials on caloric intake and food choice of 5-7-year-old children. *Appetite* 2007; **49**(1): 263-7
23. National Child Measurement Programme. Changes in children's body mass index between 2006/07 and 2015/16.: Public Health England, 2017
24. Boyland EJ, Harrold JA, Kirkham TC, Halford JC. The extent of food advertising to children on UK television in 2008. *International Journal of Pediatric Obesity* 2011; **6**(5-6): 455-61.
25. Wang Y. Cross-national comparison of childhood obesity: the epidemic and the relationship between obesity and socioeconomic status. *Int J Epidemiol* 2001; **30**(5): 1129-36.
26. Public Health England. Severe obesity in ten to eleven year olds reaches record high. In: Mills J, editor.: Public Health England; 2018
27. Wilsher, S. H., Harrison, F., Yamoah, F., Fearne, A., & Jones, A. (2016). The relationship between unhealthy food sales, socio-economic deprivation and childhood weight status: results of a cross-sectional study in England. *International Journal of Behavioral Nutrition and Physical Activity*, *13*(1), 21.
28. Sanderson, S. C., Waller, J., Jarvis, M. J., Humphries, S. E., & Wardle, J. (2009). Awareness of lifestyle risk factors for cancer and heart disease among adults in the UK. *Patient education and counseling*, *74*(2), 221-227.
29. Puhl, R., & Suh, Y. (2015). Health consequences of weight stigma: implications for obesity prevention and treatment. *Current obesity reports*, *4*(2), 182-190.
30. A Healthier Future - Scotland's Diet and Healthy Weight Delivery Plan. Edinburgh: Scottish Government, 2018.
31. Department of Health and Social Care: Global Public Health Directorate: Obesity FaN. Childhood Obesity: a plan for action. Chapter 2: HM Government, 2018
32. Swinburn BA. Obesity prevention: the role of policies, laws and regulations. *Aust New Zealand Health Policy* 2008; **5**: 12
33. WHO. World Health Organisation: Obesity and Overweight. 2017. http://www.who.int/en/news-room/fact-sheets/detail/obesity-and-overweight (accessed 24.07.18 2018).
34. Hooper L, Anderson A, Birch J, et al. Public awareness and healthcare professional advice for obesity as a risk factor for cancer in the UK: a cross-sectional survey. *Journal of Public Health* 2017.
35. Buykx P, Li J, Lovatt M, et al. An Examination of Public Attitudes Towards Alcohol Policy: University of Sheffield and Cancer Research UK, 2016
36. Bates S, Holmes J, Gavens L, et al. Awareness of alcohol as a risk factor for cancer is associated with public support for alcohol policies. *BMC Public Health* 2018; **18**(1): 688
37. Public Health England. National Diet and Nutrition Survey. 2016. https://www.gov.uk/government/collections/national-diet-and-nutrition-survey (accessed 03.01.18 2018)
38. Marketing IoS. Food Choice Survey: University of Stirling, 2007
39. Alcohol (Minimum Unit) Pricing Act 2012. http://www.legislation.gov.uk/asp/2012/4/pdfs/asp_20120004_en.pdf
40. Morley BC, Scully ML, Niven PH, et al. What factors are associated with excess body weight in Australian secondary school students? *Med J Aust* 2012; **196**(3): 189-92.
41. Department for Communities and Local Government. The English Index of Multiple Deprivation (IMD) - Guidance, 2015

42. Sugar Reduction: Achieving the 20%: Public Health England, 2017
43. Dixon HG, Scully ML, Wakefield MA, White VM, Crawford DA. The effects of television advertisements for junk food versus nutritious food on children's food attitudes and preferences. *Soc Sci Med* 2007; **65**(7): 1311-23.
44. Denny S, Lewycka S, Utter J, et al. The association between socioeconomic deprivation and secondary school students' health: findings from a latent class analysis of a national adolescent health survey. *Int J Equity Health* 2016; **15**(1): 109
45. Nestle M. Food marketing and childhood obesity--a matter of policy. *N Engl J Med* 2006; **354**(24): 2527-9.
46. Cooke LJ, Wardle J. Age and gender differences in children's food preferences. *Br J Nutr* 2005; **93**(5): 741-6.
47. MacGregor, A., Bicquelet, A., Lepps. H., Porter, L., Eadie, D., McKell, J., MacKintosh, A A., Thomas, C., Hooper, L and Vohra, J (2016) *""It's just there to trick you": A qualitative study of 11-19 year olds perceptions of food and drink marketing."* ScotCen Social Research; NatCen Social Research; Institute for Social Marketing, University of Stirling; and Policy Research Centre for Cancer Prevention, Cancer Research UK.
48. Stamatakis E, Wardle J, Cole TJ. Childhood obesity and overweight prevalence trends in England: evidence for growing socioeconomic disparities. *Int J Obes (Lond)* 2010; **34**(1): 41-7
49. McLaren L. Socioeconomic status and obesity. *Epidemiol Rev* 2007; **29**: 29-48
50. Bann D, Johnson W, Li L, Kuh D, Hardy R. Socioeconomic Inequalities in Body Mass Index across Adulthood: Coordinated Analyses of Individual Participant Data from Three British Birth Cohort Studies Initiated in 1946, 1958 and 1970. *PLoS Med* 2017; **14**(1): e1002214
51. Crawford B, Byun R, Mitchell E, Thompson S, Jalaludin B, Torvaldsen S. Socioeconomic differences in the cost, availability and quality of healthy food in Sydney. *Aust N Z J Public Health* 2017; **41**(6): 567-71
52. Campbell D. Children seeing up to 12 adverts for junk food an hour on TV, study finds. 2017. https://www.theguardian.com/society/2017/nov/28/children-seeing-up-to-12-adverts-for-junk-food-an-hour-on-tv-study-finds (accessed 23.07.18 2018)
53. Bryant T. Role of knowledge in public health and health promotion policy change. *Health Promot Int* 2002; **17**(1): 89-98
54. Raphael D and Bryant T (2006) The state's role in promoting population health: Public health concerns in Canada, USA, UK and Sweden. *Health Policy.* 78 (1) 39 - 55

For peer review only

STROBE Statement—Checklist of items that should be included in reports of ***cross-sectional studies***

	Item No	Recommendation	Page No.
**Title and abstract**	1	(*a*) Indicate the study's design with a commonly used term in the title or the abstract	1
		(*b*) Provide in the abstract an informative and balanced summary of what was done and what was found	2
**Introduction**			
Background/rationale	2	Explain the scientific background and rationale for the investigation being reported	5
Objectives	3	State specific objectives, including any prespecified hypotheses	5
**Methods**			
Study design	4	Present key elements of study design early in the paper	6
Setting	5	Describe the setting, locations, and relevant dates, including periods of recruitment, exposure, follow-up, and data collection	6
Participants	6	(*a*) Give the eligibility criteria, and the sources and methods of selection of participants	6
Variables	7	Clearly define all outcomes, exposures, predictors, potential confounders, and effect modifiers. Give diagnostic criteria, if applicable	6
Data sources/ measurement	8*	For each variable of interest, give sources of data and details of methods of assessment (measurement). Describe comparability of assessment methods if there is more than one group	6
Bias	9	Describe any efforts to address potential sources of bias	6
Study size	10	Explain how the study size was arrived at	6
Quantitative variables	11	Explain how quantitative variables were handled in the analyses. If applicable, describe which groupings were chosen and why	6, 7
Statistical methods	12	(*a*) Describe all statistical methods, including those used to control for confounding	6, 7
		(*b*) Describe any methods used to examine subgroups and interactions	7
		(*c*) Explain how missing data were addressed	Not recorded for this study as online survey was used
		(*d*) If applicable, describe analytical methods taking account of sampling strategy	6
		(*e*) Describe any sensitivity analyses	NR
**Results**			
Participants	13*	(a) Report numbers of individuals at each stage of study—eg numbers potentially eligible, examined for eligibility, confirmed eligible, included in the study, completing follow-up, and analysed	8

For peer review only - http://bmjopen.bmj.com/site/about/guidelines.xhtml

		(b) Give reasons for non-participation at each stage	This information was not collected.
		(c) Consider use of a flow diagram	-
Descriptive data	14*	(a) Give characteristics of study participants (eg demographic, clinical, social) and information on exposures and potential confounders	8, 11
		(b) Indicate number of participants with missing data for each variable of interest	6
Outcome data	15*	Report numbers of outcome events or summary measures	8
Main results	16	(*a*) Give unadjusted estimates and, if applicable, confounder-adjusted estimates and their precision (eg, 95% confidence interval). Make clear which confounders were adjusted for and why they were included	12,13,14
		(*b*) Report category boundaries when continuous variables were categorized	12,13,14
		(*c*) If relevant, consider translating estimates of relative risk into absolute risk for a meaningful time period	NR
Other analyses	17	Report other analyses done—eg analyses of subgroups and interactions, and sensitivity analyses	NR
**Discussion**			
Key results	18	Summarise key results with reference to study objectives	8
Limitations	19	Discuss limitations of the study, taking into account sources of potential bias or imprecision. Discuss both direction and magnitude of any potential bias	9,10
Interpretation	20	Give a cautious overall interpretation of results considering objectives, limitations, multiplicity of analyses, results from similar studies, and other relevant evidence	9,10
Generalisability	21	Discuss the generalisability (external validity) of the study results	9,10
**Other information**			
Funding	22	Give the source of funding and the role of the funders for the present study and, if applicable, for the original study on which the present article is based	10

*Give information separately for exposed and unexposed groups.

Note: An Explanation and Elaboration article discusses each checklist item and gives methodological background and published examples of transparent reporting. The STROBE checklist is best used in conjunction with this article (freely available on the Web sites of PLoS Medicine at http://www.plosmedicine.org/, Annals of Internal Medicine at http://www.annals.org/, and Epidemiology at http://www.epidem.com/). Information on the STROBE Initiative is available at www.strobe-statement.org.

For peer review only
